# Hemostatic conditions following autologous transfusion of fresh vs stored platelets in experimental endotoxemia: an open-label randomized controlled trial with healthy volunteers

**DOI:** 10.1016/j.rpth.2024.102612

**Published:** 2024-10-29

**Authors:** Stefan F. van Wonderen, Floor L.F. van Baarle, Anita M. Tuip-de Boer, Chantal A. Polet, Robin van Bruggen, Christie Vermeulen, Thomas R.L. Klei, Chi M. Hau, Rienk Nieuwland, Cornelis van ’t Veer, Anna L. Peters, Sanne de Bruin, Alexander P.J. Vlaar, Bart J. Biemond, Marcella C.A. Müller

**Affiliations:** 1 Laboratory of Experimental Intensive Care and Anesthesiology, Amsterdam UMC location University of Amsterdam, Amsterdam, the Netherlands; 2 Department of Intensive Care Medicine, Amsterdam UMC location University of Amsterdam, Amsterdam, the Netherlands; 3 Department of Blood Cell Research, Sanquin Blood Supply, Amsterdam, the Netherlands; 4 Department of Product and Process Development, Sanquin Blood Supply, Amsterdam, the Netherlands; 5 Laboratory for Experimental Clinical Chemistry, Amsterdam UMC location University of Amsterdam, Amsterdam, the Netherlands; 6 Amsterdam Vesicle Center, Amsterdam UMC location University of Amsterdam, Amsterdam, the Netherlands; 7 Center of Experimental and Molecular Medicine, Amsterdam UMC location University of Amsterdam, Amsterdam, the Netherlands; 8 Department of Hematology, Amsterdam UMC location University of Amsterdam, Amsterdam, the Netherlands

**Keywords:** endotoxemia, hemostasis, platelet, platelet transfusion, thromboelastography

## Abstract

**Background:**

Platelet increment is reportedly lower for maximum stored platelet concentrates (PCs) and during pyrexia, and *in vitro* function differs between fresh and stored PCs. However, little is known about the function of fresh and stored platelets during inflammation.

**Objectives:**

The aim was to study differences in hemostatic function after transfusion of fresh or stored PCs in a human model of experimental endotoxemia.

**Methods:**

Thirty-six healthy male subjects received either 2 ng/kg lipopolysaccharide (LPS) or a control (physiological saline 0.9%) and were randomly assigned to subsequently receive an autologous transfusion of either fresh (2-days-old) or stored (7-days-old) platelets, or saline control. Extracellular vesicles (EVs) were determined using flow cytometry, thrombin–antithrombin complex (TATc) was assessed using enzyme-linked immunosorbent assay, and hemostatic function was assessed using rotational thromboelastometry (ROTEM).

**Results:**

LPS infusion caused a marked increase in TATc, EVs and fibrinolysis. Thromboelastometry data revealed that following infusion of LPS, subjects exhibited in general a hypocoagulable state compared with those not receiving LPS. Platelet transfusions led to a reduced clotting time and an augmentation in clot strength, indicated by maximum clot firmness, solely among subjects undergoing endotoxemia. There were no significant differences in TATc or amount of EVs release after transfusion of fresh or stored platelets.

**Conclusion:**

A significant increase in TATc and EVs as well as a difference in hemostatic function after endotoxemia were observed. During endotoxemia, platelet transfusion resulted in enhanced coagulation and hemostatic function; however, no substantial differences were observed between transfusion of fresh or stored PCs.

## Essentials

• Inflammation and platelet storage lesion affecting platelet concentrate quality.
• We compared the effect of fresh with that of stored transfused platelet concentrates on hemostasis in human subjects.
• During inflammation, subjects were generally hypocoagulable.
• Transfusion during inflammation induced procoagulant effects with no difference by storage time.

## 1 Introduction

Platelets play a central role in hemostasis, which is why platelet transfusions are frequently used to prevent or treat hemorrhage in patients with low platelet count or platelet dysfunction [1,2]. The most commonly used indicators for successful transfusion are the platelet count increment or the corrected count increment (CCI) [3,4]. The CCI is frequently reported to decrease in patients with fever and to increased platelet concentrate (PC) storage duration [[5], [6], [7], [8]]. Storage of platelets, even in optimal blood bank conditions, results in increasing expression of platelet activation markers (eg, P-selectin [CD62p] and phosphatidylserine), alterations within storage media, and the release of biologic response modifiers (eg, soluble CD40 ligand and bioactive lipids) and extracellular vesicles (EVs). This phenomenon is known as the platelet storage lesion and increases with storage duration [6,9,10].

Besides their classic role in hemostasis, platelets have recently been recognized as actors in the immune system [[11], [12], [13], [14]]. Interplay between inflammation and coagulation occurs on various levels, and here too, platelets have a pivotal role [[11], [12], [13], [14]]. Platelet–leukocyte interaction through CD62p and CD40 signaling is important in attracting monocytes and neutrophils to sites of vascular injury and in promoting leukocyte adhesion to the injured endothelium [11,14]. On the other hand, the consequently activated leukocytes shed EVs that expose phosphatidylserine and that may express tissue factor in the case of monocyte-derived vesicles [15,16]. Tissue factor initiates coagulation while phosphatidylserine exposure provides a docking place for the key coagulation factors on cells and vesicles, which lead to thrombin formation [17]. Even low concentrations of thrombin activate platelets and trigger fibrin formation [15,18,19].

Similarly to leukocyte-driven thrombin generation leading to platelet activation and consumption, extended PC storage duration also causes platelet activation [20]. However, the impact of PCs with extended storage duration on hemostatic conditions, particularly in comparison with fresh platelets, as well as during inflammation, remains unclear. Therefore, the aim was to study differences in coagulation, EVs release, and CCI following transfusion of either fresh or stored autologous platelets in healthy volunteers exposed to a model of controlled endotoxemia using lipopolysaccharide (LPS) or a saline control.

## 2 Methods

This study is part of a larger study on the influence of storage time of platelets in a 2-hit healthy volunteer model on transfusion-related acute lung injury (TRALI), including the full cohort, with a non–LPS-exposed control group [20]. The sample size was determined based on the primary outcome of the entire study, which included bronchoalveolar lavage fluid protein content, and was described previously [20]. The study protocol was reviewed by the Amsterdam University Medical Centers Location University of Amsterdam Medical Ethical Committee and was performed in accordance with the Declaration of Helsinki and Good Clinical Practice guidelines. All volunteers provided written informed consent prior to study enrollment. The study is registered at the World Health Organization—International Clinical Trials Registry Platform: NL-OMON26852.

### 2.1 Subjects, design, and randomization

We included 36 male subjects aged 18 to 35 years who were screened prior to participation and found to be healthy. Subjects were not allowed to participate in any other intervention trial during the 3 months prior to and after the study, while any previous participation in a LPS trial was a reason for exclusion to receive LPS. The first 18 subjects were allocated to receive LPS first whereas the last 18 subjects received an equal volume of physiological saline (0.9% [weight/volume] sodium chloride) as control. Subjects receiving LPS were eligible to participate again in the control group with a minimum time interval of at least 1 year between inclusions, allowing them to serve as their own controls. Per study arm, subjects were randomly allocated to receive either fresh autologous PC (stored for 2 days), stored autologous PC (stored for 7 days), or an equal volume of physiological saline resulting in 6 different groups (Supplementary Figure S1). Treatment allocation concealment was achieved by generating a randomization sequence using R-Studio (version 4.3.2, RStudio Team). The sequence was placed in consecutively numbered opaque envelopes, which were opened only after the inclusion of each subject. An open-label randomized trial was conducted given that subjects were required to donate either 2 or 7 days before the experiment. Additionally, subjects were not blinded whether they received saline or a PC transfusion.

### 2.2 PCs

All subjects donated 1 unit of apheresis PC (volume, 300 mL; platelet count, ±300 × 10^9^/L) either 2 or 7 days before the experiment, including those allocated to the saline control group, half of whom donated 2 days and half of whom donated 7 days before the experiment. PCs were collected and stored according to Dutch Blood Bank standards and stored in 100% platelet additive solution E. On the day of the experiment, 50 mL of the PC was labeled with NHS-biotin (EZ-Link, Thermo Fisher Scientific), according to a previously published protocol, and returned to the storage bag [21]. The biotin label allowed us to trace the transfused platelets over time and will be presented elsewhere.

### 2.3 Endotoxemia model

Subjects were admitted to the study ward at 7:00 am following an overnight fast. From admission to discharge, they stayed on bed rest and continued fasting throughout the study duration. The subjects were infused with 2 ng/kg *Escherichia coli* LPS (National Institutes of Health Clinical Center) as a bolus injection or an equal volume of physiological saline after 1 L 0.9% (weight/volume) sodium chloride prehydration. Two hours after LPS administration, subjects received either the fresh or stored autologous PC or saline, infused over 50 minutes. Blood was drawn from an indwelling arterial catheter prior to LPS administration, prior to PC administration, 10 minutes after completion of the transfusion, and 1, 3, 4, and 5 hours thereafter. Subjects were then discharged home and returned 48 hours after transfusion for a venous blood sample (Supplementary Figure S2).

### 2.4 Platelet count and platelet count increment

Platelet counts were measured during the screening, before LPS, before transfusion, and 1, 3 and 5 hours after completion of the transfusion using a hematology analyzer (normal range, 150-400 × 10^9^/L; XN-9000, Sysmex). The difference (Δ) between the platelet count at screening and at admission to the study ward was calculated to assess the effect of the donation. The platelet increment was determined by the difference in platelet count before and 1 hour after the transfusion, and the CCI was calculated as platelet count increment per microliter × body surface area (m^2^)/unit content [3].

### 2.5 Thrombin–antithrombin complex

Platelet poor plasma was obtained from ethylenediaminetetraacetic acid anticoagulated blood (1750*g*, 10 minutes) and stored at −80 °C until batch analysis. Thrombin–antithrombin complex (TATc) levels were determined before LPS, before transfusion and 1 and 5 hours after transfusion, using enzyme-linked immunosorbent assay (Affinity Biologicals).

### 2.6 EVs

Platelet poor plasma was obtained prior to LPS infusion, prior to transfusion, and 1 and 5 hours after transfusion. Citrated blood was centrifuged twice (2500*g*, 15 minutes); supernatant plasma was snap-frozen in liquid nitrogen and stored at −80 °C until batch analysis. CD61-BV421 positive platelet-derived (BD Biosciences), CD45-allophycocyanin positive leukocyte-derived (BioLegend), and CD235-phycoerythrin positive erythrocyte-derived (Dako Agilent) vesicles were identified from the samples. Vesicles were further determined using lactadherin-fluorescein isothiocyanate (Prolytix), and biotinylated platelet vesicles were identified using streptavidin-allophycocyanin (BD Biosciences) [22]. EVs concentration was determined with a calibrated flow cytometry (A60-Micro, Apogee Flow Systems) using settings optimized for detection of EVs. The EV concentration describes the number of particles that are positive at the relevant fluorescent detectors with a diameter <1000 nm (Rosetta Calibration v2.05, Exomtery BV). Custom-built software (MATLAB R2020b, MathWorks Inc) was used for data calibration and analysis (Supplementary Text S1).

### 2.7 Thromboelastometry

Whole blood rotational thromboelastometry measurements were performed using citrated blood on a ROTEM delta machine (Werfen). Thromboelastometry measurements included EXTEM, INTEM, and FIBTEM and were done prior to LPS infusion, prior to transfusion, 10 minutes, 4 hours, and 48 hours after transfusion. All measurements were allowed to run for 90 minutes. Collected measures include clotting time (CT), clot formation time (CFT), α angle, the amplitude after 10 minutes (A10), maximum clot firmness (MCF), and maximum lysis.

### 2.8 Statistical analysis

This study is part of a larger study on the effect of fresh vs stored PC on the development of pulmonary inflammation and markers of TRALI [20]. As the primary endpoint of the primary study was protein leakage in bronchoalveolar lavage fluid, the results of this study must therefore be considered exploratory. Data were assessed for normality, homogeneity of variance and sphericity both visually and using the Shapiro–Wilk test, Levene’s test and Mauchly’s test, respectively. Parametric data are expressed as mean (±SD) and nonparametric data as median (IQR). Between-group differences were analyzed using analysis of variance, a Kruskal–Wallis test, Student’s *t*-test or Wilcoxon signed rank test as appropriate. To assess the impact of the stimulation (LPS or control), the difference before and after stimulation was calculated and then tested. Repeated measurements were analyzed using analysis of variance repeated measurements with an interaction term for time and intervention (fresh platelets, stored platelets, or saline), and using Greenhouse–Geisser corrections as appropriate. A *P* value of <.05 was considered to be statistically significant. Data were analyzed using R-Studio (version 4.3.2) [23].

## 3 Results

### 3.1 Endotoxemia

Between April 2018 and July 2023, 36 subjects were included in the study. In total, 34 out of 36 subjects were included for further analysis (Supplementary Figure S1). Twelve subjects participated twice. One protocol violation occurred when a platelet transfusion, stored for 7 days, was inadvertently subjected to radiation treatment, following the standard therapy for allogeneic apheresis PCs by the Blood Bank. Another subject was excluded for analyses due to abnormally high cytokine levels at baseline. As previously described, LPS infusion in our study led to a significant increase in temperature, heart rate, leukocyte count, and levels of inflammatory markers (interleukin-6, C-X-C-motif ligand 8, and tumor necrosis factor α), and a significant decrease in mean arterial pressure and oxygen saturation [20]. Levels of inflammatory markers showed a peak 2 hours after LPS infusion and changes in vital parameters were at a maximum approximately 4 hours after LPS infusion. Baseline characteristics were similar between groups (Table).

**Table:** Baseline demographics.

Variable	Overall (*N* = 34)	Lipopolysaccharide			Control			*P* value
		Fresh platelets (*n* = 6)	Stored platelets (*n* = 6)	Saline (*n* = 5)	Fresh platelets (*n* = 6)	Stored platelets (*n* = 5)	Saline(*n* = 6)	
Age (y)	25 (23-28)	23 (23-25)	27 (26-29)	25 (21– 26)	25 (24-27)	24 (21-24)	26 (24– 29)	.48
BMI (kg/m^2^)	23.5 (22.7-24.6)	23.4 (22.7-23.5)	26.3 (23.5-28.3)	24.0 (22.1-24.3)	23.5 (22.8-24.3)	23.2 (23.1-27.2)	23.9 (23.3-24.3)	.84
Laboratory values at screening								
Platelet count (×10^9^/L)	280 (56)	295 (83)	261 (42)	270 (38)	295 (83)	291 (41)	269 (36)	.86
Laboratory values at admission								
Platelet count (×10^9^/L)	199 (39)	201 (57)	181 (40)	211 (41)	206 (45)	216 (22)	187 (22)	.66
Hemoglobin (g/dL)	8.0 (7.6-8.4)	8.3 (8.2-8.4)	7.9 (7.7-8.4)	7.9 (7.6-8.0)	8.2 (7.6-8.9)	8.2 (8.1-8.7)	8.0 (7.9-8.0)	.56
Leukocytes (×10^9^/L)	5.2 (1.1)	5.8 (1.2)	4.6 (1.2)	4.8 (1.3)	5.7 (1.4)	5.4 (0.5)	4.9 (0.4)	.26
TATc complex (pg/mL)	5.7 (2.7-9.9)	9.1 (3.8-15.7)	7.2 (5.4-14.5)	4.9 (4.7-5.1)	4.0 (2.2-8.9)	6.2 (6.2-10.3)	4.1 (2.7-5.1)	.38
EXTEM								
Clotting time (s)	70 (8.2)	68 (7.0)	65 (9.8)	69 (6.9)	78 (7.9)	72 (4.6)	69 (8.3)	.13
Clot formation time (s)	105 (97-116)	112 (107-115)	108 (96-117)	102 (93-106)	103 (99-110)	100 (88-107)	106 (99-131)	.79
α angle (°)	69 (2.9)	68 (1.2)	68 (4.9)	69 (3.4)	69 (1.8)	70 (2.7)	68 (3.1)	.85
Amplitude at 10 min (mm)	49 (3.5)	47 (1.8)	49 (5.0)	49 (3.9)	50 (2.2)	51 (3.1)	48 (4.2)	.49
Maximum clot firmness (mm)	57 (3.1)	55 (1.6)	57 (3.7)	56 (4.2)	58 (2.0)	59 (2.2)	57 (3.9)	.32
Maximum lysis (%)	16 (3.1)	16 (4.6)	16 (3.3)	18 (2.4)	15 (4.0)	16 (2.3)	15 (1.7)	.82
INTEM								
Clotting time (s)	190 (176-204)	190 (177-197)	183 (165-192)	178 (150-181)	191 (182-203)	203 (201-213)	190 (166-204)	.31
Clot formation time (s)	84 (78-94)	88 (83-93)	83 (77-101)	86 (79-99)	81 (78-85)	80 (79-94)	83 (77-106)	.95
α angle (°)	73 (2.2)	74 (1.1)	73 (2.9)	73 (2.1)	74 (1.7)	73 (2.0)	73 (3.3)	.96
Amplitude at 10 min (mm)	49 (2.9)	48 (1.6)	49 (4.6)	49 (3.3)	51 (1.3)	50 (2.6)	49 (3.1)	.61
Maximum clot firmness (mm)	55 (3.2)	54 (2.5)	55 (4.1)	54 (4.2)	57 (2.5)	56 (2.0)	56 (3.0)	.40
Maximum lysis (%)	15 (13-17)	16 (14-18)	15 (14-17)	16 (16-17)	14 (9-17)	13 (13-17)	14 (13-15)	.76
FIBTEM								
Clotting time (s)	65 (8.6)	61 (9.6)	63 (10.8)	66 (9.6)	64 (10.1)	66 (1.3)	70 (6.5)	.61
Maximum clot firmness (mm)	11 (2.7)	11 (1.2)	12 (4.5)	9 (3.1)	11 (1.5)	13 (3.0)	11 (2.0)	.53
α angle (°)	63 (6.7)	58 (3.1)	72 (3.5)	70 (6.4)	64 (3.5)	60 (5.9)	61 (9.5)	.03
Maximum lysis (%)	2 (0-5)	0 (0-3)	6 (4-11)	1 (0-2)	3 (1-3)	2 (0-3)	4 (0-7)	.33

All variables are displayed as count (%) for categorical data, median (first to third quartile) for nonparametric data and mean (SD) for parametric data.

BMI, body mass index; TATc, thrombin–antithrombin complex.

### 3.2 Platelet count and CCI

**Figure 1:**
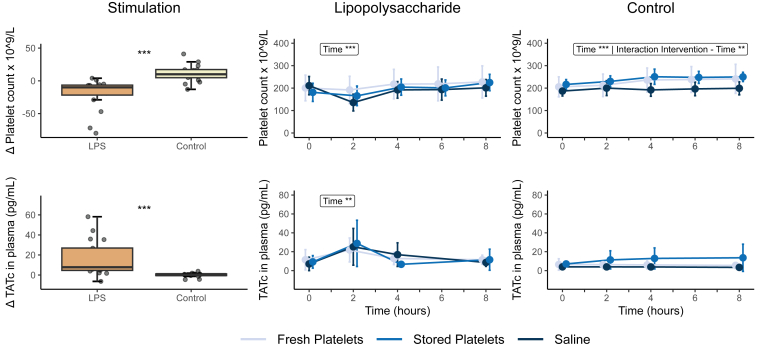
Platelet count and thrombin–antithrombin (TATc) levels Δ and over time. Platelet count measurements at different time points during the experiment, showing a decrease 2 hours after infusion of lipopolysaccharide (LPS). There is an increase 2 hours after autologous platelet transfusion of both fresh and stored platelets in the control group. TATc levels increase after LPS infusion, whereas TATc levels remained stable in the control group. Data were analyzed with Student’s *t*-test or Wilcoxon signed rank test as appropriate and analysis of variance repeated measurements, with main effects for intervention and time, and an interaction effect: ∗*P* < .05; ∗∗*P* < .01; ∗∗∗*P* < .0001. Lipopolysaccharide (2 ng/kg) was administered immediately after time point 0 hours, and the transfusion was administered immediately after time point 2 hours.

The mean platelet count at screening was 280 × 10^9^/L (±56 × 10^9^/L), whereas the baseline platelet count (before LPS or placebo administration) in all subjects was 199 × 10^9^/L (±39 × 10^9^/L) (Table). The difference between the platelet count at screening and at admission to the study ward was 81 × 10^9^/L (±33 × 10^9^/L) (Supplementary Figure S3). No significant differences were found in platelet counts or the difference in platelet count after the donation between subjects donating 2 days or 7 days before the study. The platelet count significantly decreased temporarily 2 hours after LPS infusion (Figure 1). In the LPS-exposed group, the platelet counts increased after transfusion of either fresh platelets or stored platelets and after administration of saline. In the control group that did not receive LPS, the platelet count increased after the fresh platelet transfusion from 212 × 10^9^/L (±50 × 10^9^/L) to 236 × 10^9^/L (±53 × 10^9^/L) and from 230 × 10^9^/L (±25 × 10^9^/L) to 251 × 10^9^/L (±34 × 10^9^/L) after transfusion of stored platelets. The platelet count slightly decreased from 200 × 10^9^/L (±33 × 10^9^/L) to 192 × 10^9^/L (±28 × 10^9^/L) after receiving saline instead of platelets. There was a notable rise in platelet count over time following a PC transfusion compared with saline administration in the control group. There were no differences in increment (*P* = .56) and CCI (*P* = .75) between the 4 groups (LPS/control and fresh/stored platelets) receiving a PC (Supplementary Figure S4). The CCI for fresh and stored platelets in the control group were 16,163 (±8785) and 13,356 (±15,550), respectively. The CCI in subjects receiving LPS were 17,324 (±7910) for fresh platelets and 27,116 (±25,563) for stored platelets.

### 3.3 TATc

In the LPS-exposed group, the median plasma TATc levels significantly increased to a peak value of 25.3 pg/mL (±18.4 pg/mL), 2 hours after LPS infusion when compared with subjects receiving the control (*P* < .0001). Two hours after the transfusion, TATc levels had returned to the baseline value. In the control group, not receiving LPS, TATc levels remained stable over time irrespective of the administration of either saline, fresh, or stored platelets (Figure 1).

### 3.4 EVs

**Figure 2:**
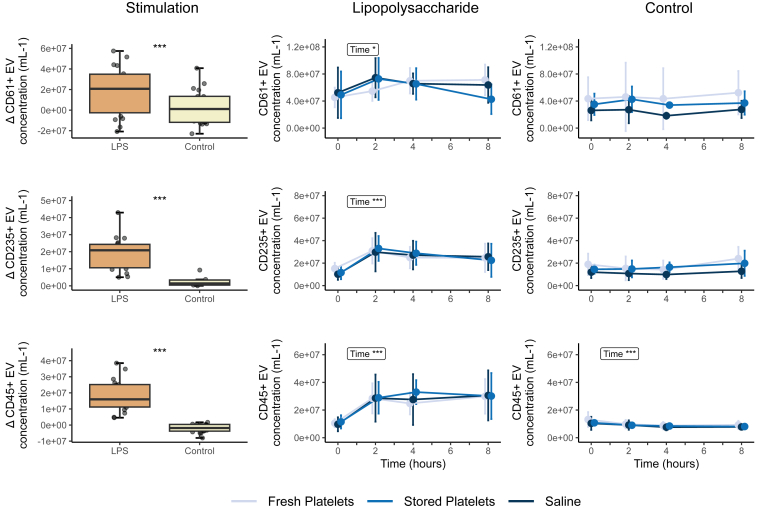
Circulating levels of extracellular vesicles (EVs) Δ and over time. Extracellular vesicles increased after lipopolysaccharide (LPS) infusion but not after infusion of the control. Extracellular vesicles were analyzed with Student’s *t*-test or Wilcoxon signed rank test as appropriate and analysis of variance repeated measurements, with main effects for intervention and time, and an interaction effect: ∗*P* < .05; ∗∗*P* < .01; ∗∗∗*P* < .0001. Lipopolysaccharide (2 ng/kg) was administered immediately after time point 0 hours, and the transfusion was administered immediately after time point 2 hours.

The concentrations of CD61-positive (platelet-derived), CD235-positive (erythrocyte-derived), and CD45-positive (leukocyte-derived) EVs increased significantly 2 hours after LPS infusion (Figure 2). This increase was not observed after giving saline instead of LPS. There were no differences between transfusion groups in amount of increase for any type of EVs. The increase in CD61-positive (platelet-derived) EVs in response to LPS infusion was, however, less pronounced than the increase in other types of EVs, while their concentration at baseline was already higher than erythrocyte- and leukocyte-derived vesicles. The proportion of biotin-labeled CD61-positive vesicles rose to 0.27% (±0.18%) and 0.33% (±0.30%) following transfusion of fresh and stored platelets in the LPS-exposed group, respectively. In the control group, the percentage of biotin-labeled CD61-positive vesicles increased to 0.36% (±0.26%) and 0.35% (±0.26%) after transfusion of fresh and stored platelets. There were no significant differences in biotin-labeled CD61-positive vesicles between fresh and stored platelets. The biotin-labeled CD61-positive vesicles slightly decreased 5 hours after transfusion in the non-LPS group in both fresh and stored platelets (Supplementary Figure S5).

### 3.5 Thromboelastometry

**Figure 3:**
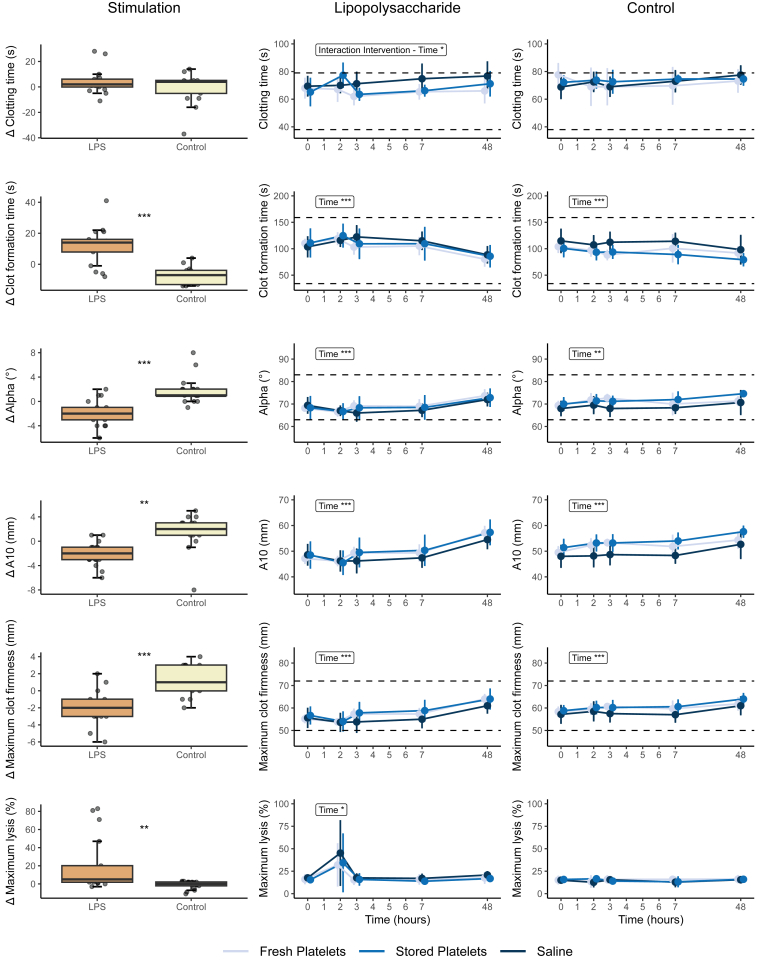
EXTEM rotational thromboelastometry measurements Δ and over time. EXTEM thromboelastometry was analyzed with Student’s *t*-test or Wilcoxon signed rank test as appropriate and analysis of variance repeated measurements, with main effects for intervention and time, and an interaction effect: ∗*P* < .05; ∗∗*P* < .01; ∗∗∗*P* < .0001. Normal reference values are indicated with horizontal striped lines. Lipopolysaccharide (LPS; 2 ng/kg) was administered immediately after time point 0 hours, and the transfusion was administered immediately after time point 2 hours. A10, amplitude after 10 minutes.

**Figure 4:**
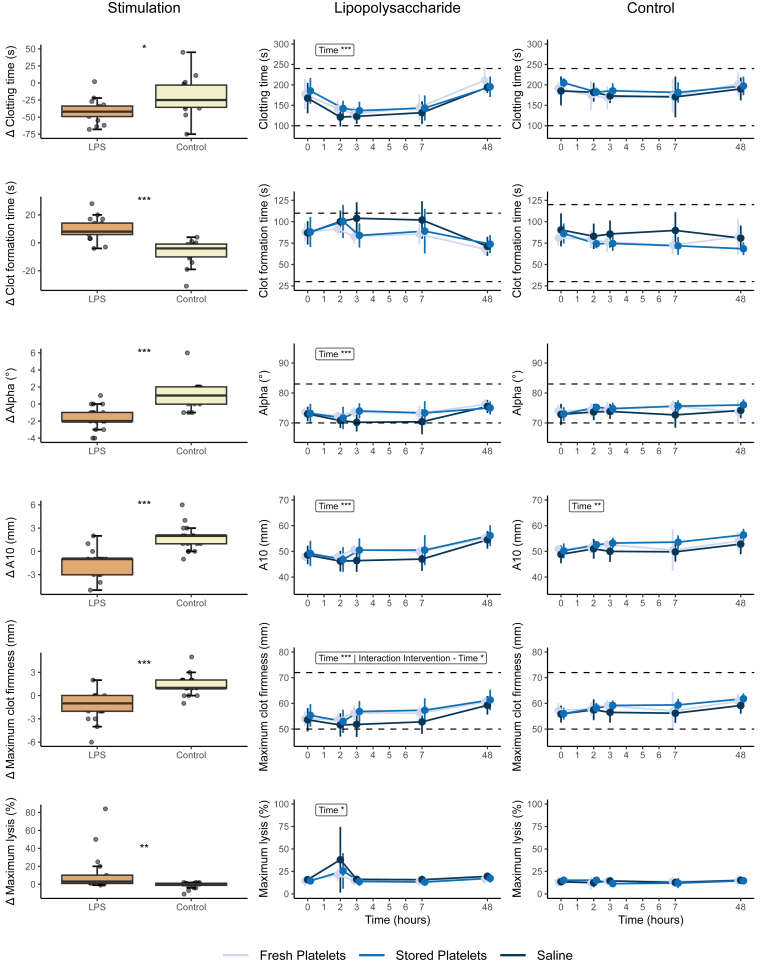
INTEM rotational thromboelastometry measurements Δ and over time. INTEM thromboelastometry data were analyzed with Student’s *t*-test or Wilcoxon signed rank test as appropriate and analysis of variance repeated measurements, with main effects for intervention and time, and an interaction effect: ∗*P* < .05; ∗∗*P* < .01; ∗∗∗*P* < .0001. Normal reference values are indicated with horizontal striped lines. Lipopolysaccharide (LPS; 2 ng/kg) was administered immediately after time point 0 hours, and the transfusion was administered immediately after time point 2 hours. A10, amplitude after 10 minutes.

**Figure 5:**
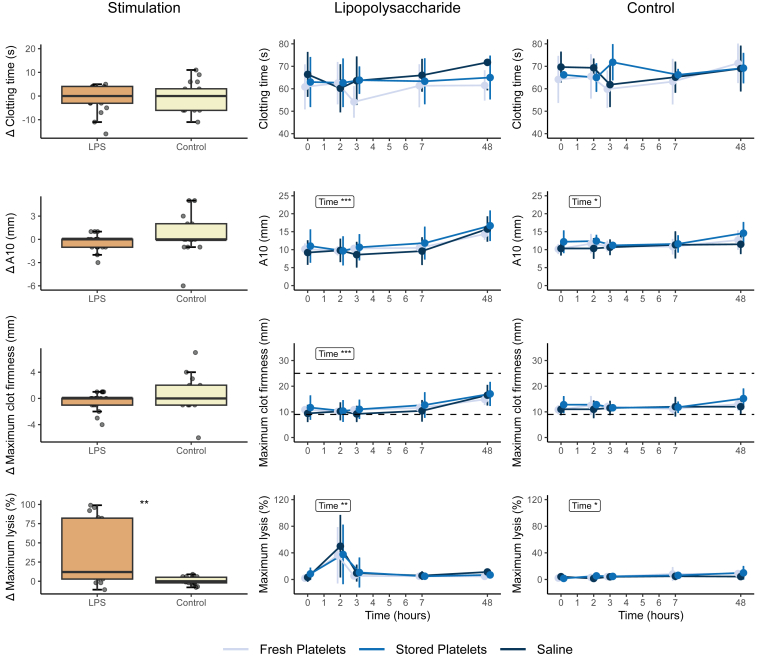
FIBTEM rotational thromboelastometry measurements Δ and over time. FIBTEM thromboelastometry data were analyzed with Student’s *t*-test or Wilcoxon signed rank test as appropriate and analysis of variance repeated measurements, with main effects for intervention and time, and an interaction effect: ∗*P* < .05; ∗∗*P* < .01; ∗∗∗*P* < .0001. Normal reference values are indicated with horizontal striped lines. Lipopolysaccharide (LPS; 2 ng/kg) was administered immediately after time point 0 hours, and the transfusion was administered immediately after time point 2 hours. A10, amplitude after 10 minutes.

There were significant changes in response to LPS infusion in all thromboelastometry parameters, generally showing a hypocoagulable and hyperfibrinolytic pattern. EXTEM and INTEM CFT significantly increased after LPS infusion. A reduction in EXTEM MCF, α angle, and A10 was observed, alongside a decrease in INTEM CT, MCF, α angle, and A10 (Figures 3 and 4). Notably, maximum lysis increased in EXTEM, INTEM, and FIBTEM 2 hours after LPS infusion and then returned to baseline values from 4 hours after LPS infusion onward (Figure 3, Figure 4, Figure 5). After transfusion of both fresh and stored platelets the EXTEM and INTEM CFT that had initially increased after LPS infusion, nonsignificantly returned to baseline values immediately after transfusion. Similarly, there was a small but nonsignificant increase in α angle, A10, and MCF immediately after transfusion in both transfusion groups. These changes were not observed in the saline control group (Figures 3 and 4). An interaction between the intervention and time was identified exclusively within the EXTEM CT and INTEM MCF of subjects who received LPS prior to transfusion. This interaction indicates a reduction in CT, particularly notable in stored platelets, and an improvement in the strength of the fibrin and platelet clot after receiving a platelet transfusion (Figures 3 and 4).

## 4 Discussion

This study was part of a larger project examining the impact of platelet storage time on TRALI in a 2-hit healthy volunteer model, including the full cohort and a control group that was not exposed to LPS. We report the results of an open-label randomized controlled substudy, an experimental endotoxemia model, and autologous platelet transfusion, where fresh and stored PCs were transfused in healthy volunteers at the peak of inflammation-induced coagulopathy. In response to infusion of LPS, our study subjects experienced a temporary signs of consumption coagulopathy as expressed by a decrease in platelet count and concomitantly a hypocoagulable state expressed by prolonged coagulation times, reduction of MCF, and an increased fibrinolysis in thromboelastometry analysis. Hereby, we found increased levels of TATc and circulating EVs. Additionally, EXTEM CT decreased after transfusion, particularly with stored platelets. There were no substantial differences between transfusion of either fresh or stored platelets with regard to CCI, TATc levels, EV release, and thromboelastometry parameters. Following platelet transfusion in subjects receiving LPS, there were significant signs of procoagulant effects observed, including increased MCF and decreased CT, compared with transfusion in the saline control group.

Interestingly, in the LPS-exposed group, both the transfusion of PCs and the administration of saline at the peak of inflammation resulted in an increased platelet count. This suggests that the decreasing effect of LPS after its peak had a greater influence on the platelet count than the interventions themselves. In previous clinical studies, a decreased CCI 1 and 24 hours after transfusion was observed for stored platelets compared with fresh platelets, and fever and infection were associated with a reduction of the posttransfusion platelet increment [[5], [6], [7], [8]]. In this study, we were not able to find differences in the CCI between the specific groups. This contrasting finding could be explained by the rather small study sample size, the baseline platelet count being too high, or the lower severity of the endotoxemia-induced illness compared with the patients in those studies. On the other hand, we did find differences in Δ platelet count between the LPS and saline groups, which is in line with previous studies showing lower CCI and/or posttransfusion recovery during inflammation.

Partly consistent with our findings, elevated TATc values have been observed not only in patients with sepsis but also following platelet transfusion in specific populations [24,25]. Indeed, we observed increased TATc levels after LPS, but transfusion did not result in a further increase. In the control group not receiving LPS, we also found no differences TATc values between the interventions, which differs from observations in patients with liver disease–associated thrombocytopenia, who exhibited a 26% increase in TATc levels following a platelet transfusion [25]. However, as noted by the authors, the patients who exhibited the largest percentage increases in TATc levels had their posttransfusion blood samples taken after a procedure and not directly after the transfusion, which likely contributed to the rise in plasma TATc levels [25].

Unexpectedly, transfusion of neither fresh nor stored platelets led to increased levels of circulating platelet-derived EVs. Previous studies of platelet transfusion in severely thrombocytopenic patients resulted in an increase in platelet-derived EVs to peak levels more than 30-fold lower than the baseline levels in our healthy subjects. This indicates that most circulating EVs are derived from the native platelets, masking any potential differences between fresh and stored platelets [26]. However, in line with our findings, platelet-derived EVs in patients with sepsis were significantly higher than normal controls [27], underlining the enhanced activation of native platelets due to inflammation.

The observed alterations in thromboelastometry measurements partially align with results from previous studies on sepsis and experimental endotoxemia. A prior investigation involving healthy volunteers demonstrated a decline in CT within 6 hours after endotoxemia, which is consistent with our findings indicating a significant reduction in INTEM CT following LPS infusion [28]. Notably, while CFT remained relatively stable over time in the previous study with healthy volunteers, we observed an increase in CFT in both EXTEM and INTEM, indicative of a hypocoagulable state induced by endotoxemia [28]. Furthermore, LPS infusion previously showed no discernible effect on blood clot strength, as measured by MCF [28]. However, our study revealed a reduced MCF among subjects during endotoxemia, in line with the declining platelet count, indicating a consumption coagulopathy. Our results align with thromboelastometry data obtained from septic patients with disseminated intravascular coagulation, also revealing prolonged CT formation and decreased MCF [29]. Consistent with previous findings, a brief episode of hyperfibrinolysis was observed following LPS infusion [[28], [29], [30], [31], [32], [33], [34], [35]]. The increase in clot lysis 2 hours after LPS administration parallels previous reports on experimental endotoxemia that demonstrated an increase in fibrinolysis 2 hours after LPS administration that is reduced by the increased levels of plasminogen activator inhibitor 1 inhibiting the increased fibrinolytic activity [36,37].

Both fresh and stored platelets had similar effects on thromboelastometry parameters. Platelet transfusion, both fresh and stored, notably reduced CT and enhanced MCF, particularly among subjects experiencing endotoxemia. These observations underline that platelet transfusion in a state of inflammation has more pronounced effects on coagulation compared with noninflammatory conditions. This further supports a cautious platelet transfusion strategy in patients with inflammation-induced consumption coagulopathy when platelets are solely transfused to counteract of low platelet count. To our knowledge, there are no previously published reports on thromboelastometry-assessed coagulation after transfusion of fresh vs stored platelets; however, *in vitro* studies have shown conflicting results of thromboelastography-assessed coagulant function in longer stored platelets [[38], [39], [40], [41]].

There are some important limitations to the observations presented in this study. First, this study was part of a larger study examining the impact of platelet storage time, and there has been no adjustment for multiplicity. These results must therefore be considered exploratory. Nonetheless, the research questions were determined prior to the study, and all data were collected prospectively. Subjects were not randomly assigned to receive either LPS or saline, which could potentially lead to unbalanced groups. However, baseline demographics were similar across all groups in the study. The healthy volunteers in this study had a temporary and “mild thrombocytopenia” caused by the LPS-induced inflammatory response, which potentially limits the translation of our results to patients with ongoing severe thrombocytopenia, for instance, in the case of disseminated intravascular coagulation, and even more so in the case of hypoproliferative thrombocytopenia. The hypocoagulable state after LPS infusion was likely due to the effect on the subjects’ native platelets. We cannot preclude that effects between fresh and stored platelets would become apparent in patient with thrombocytopenia when the ratio of transfused platelets to native platelets is higher than in our study population. The relatively low ratio of transfused platelets to native platelets may have led to false-negative outcomes regarding the effect of transfused PCs, as the transfused platelets have been diluted and outnumbered by the native platelets. Nonetheless, the trend toward improved thromboelastometry-assessed coagulation in both transfusion groups compared with that in the saline control group corresponds to changes observed in thrombocytopenic patients and surgical patients receiving prophylactic platelet transfusion [[42], [43], [44], [45], [46]]. There are important differences between the experimental endotoxemia model and actual sepsis. However, the changes in coagulation parameters observed here and in other studies of experimental endotoxemia, although short-lived, correspond with the direction of change seen in septic patients. We did not collect data on the race and ethnicity of the volunteers due to national regulations. However, it is important to note that individuals of different racial backgrounds may respond differently to coagulation changes. Future research should consider including diverse racial and ethnic groups to account for these potential variations. Lastly, the sample size might have been insufficient to discern disparities between fresh and stored platelets, and exploring alternative methods to assess coagulation status, such as bleeding time to measure *in vivo* platelet function at the time of the test, could possibly have exposed now unseen differences. An important strength of this study is the randomized and controlled setting, with standardized endotoxemia, platelet products, and blood sampling. Especially, the autologous nature of this study allowed us to fairly compare fresh and stored PCs without interference of donor variability. And the fact that a large proportion of subjects acted as their own control allowed us to more robustly find differences between the endotoxemic phenotype and control. Furthermore, it stands out as the largest study of its kind to date. Moreover, the wide array of outcome measures has allowed us to investigate differences between fresh and stored platelets from different angles.

The results of this study indicate no significant differences in coagulant function between fresh and stored platelets, also in a controlled experimental endotoxemia setting. However, in inflammation, platelet transusion had more outspoken procoagulant effects. Without inflammation, solely, an increase in platelet count was observed after transfusion of PCs. From a clinical perspective, this suggests that the storage duration of platelets may be less critical when transfused during inflammation, although PCs can induce some procoagulant effects. Therefore, physicians should exercise caution when transfusing platelets to patients with sepsis who are already in a procoagulant state, as it may induce further activation. Further research is needed to refine the platelet transfusions strategies.

In conclusion, our study revealed no substantial differences between fresh and stored platelets in terms of their impact on thromboelastometry-assessed coagulant function after autologous platelet transfusion at the peak of inflammation in a controlled human model of experimental endotoxemia. However, a platelet transfusion during inflammation induced procoagulant effects; therefore, caution should be exercised in patients with sepsis.
