# Supplementary material for: Hemostatic conditions following autologous transfusion of fresh vs stored platelets in experimental endotoxemia: an open-label randomized controlled trial with healthy volunteers
Source: Res Pract Thromb Haemost. 2024 Oct 29;8(8):102612. doi: 10.1016/j.rpth.2024.102612 (PMC11616504; doi:10.1016/j.rpth.2024.102612)

Figures

Supplemental materials to:

Hemostatic Conditions Following Autologous Transfusion of Fresh versus Stored Platelets in Experimental Endotoxemia: an Open-label Randomized Controlled Trial with Healthy Volunteers

Stefan F. van Wonderen, Floor L.F. van Baarle, Anita M. Tuip-de Boer, Chantal A. Polet, Robin van Bruggen, Christie Vermeulen, Thomas R.L. Klei, Chi M. Hau, Rienk Nieuwland, Cornelis van 't Veer, Anna L. Peters, Sanne de Bruin, Alexander P.J. Vlaar, Bart J. Biemond, Marcella C.A. Müller

TABLE OF CONTENTS

Figure S1. Consort diagram 3

Figure S2. Overview of study procedures 4

Figure S3. Change in platelet count between screening and admission. 5

Figure S4. Platelet count increment and corrected count increment (CCI) 6

Figure S5. Circulating levels of extracellular vesicles 7

Figure S1. Consort diagram. Study subjects flow through enrollment, allocation, follow-up and analyses.

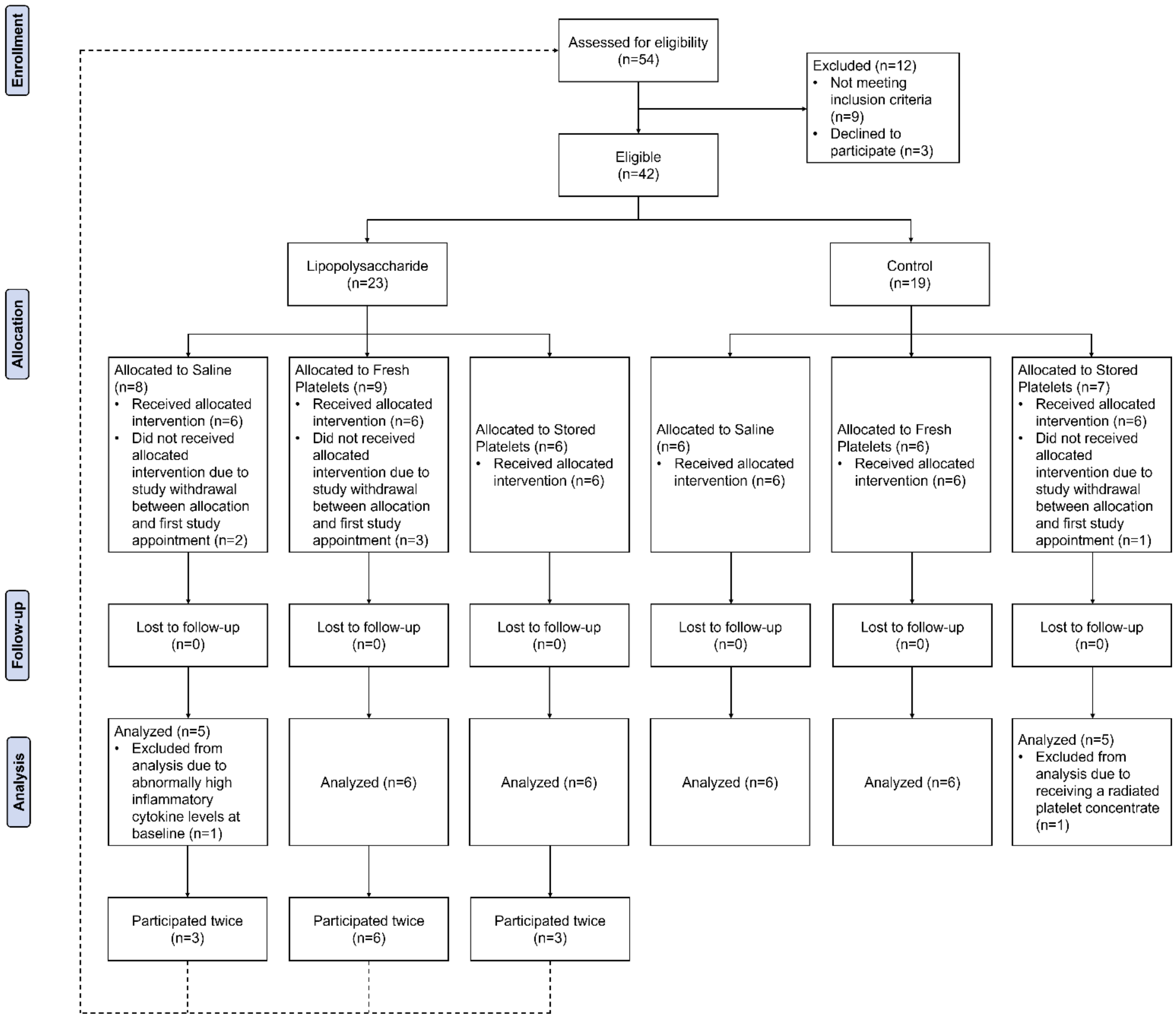

Figure S2. Overview of study procedures. The study's time points are centrally presented, encompassing flow cytometry extracellular vescicles and enzyme-linked immunosorbent assay (ELISA) Thrombin–antithrombin complex (TATc) determination at the top, and rotational thromboelastometry (using ROTEM®) analyses at the bottom. Other abbreviations: LPS, Lipopolysaccharide. *Created with BioRender.com*

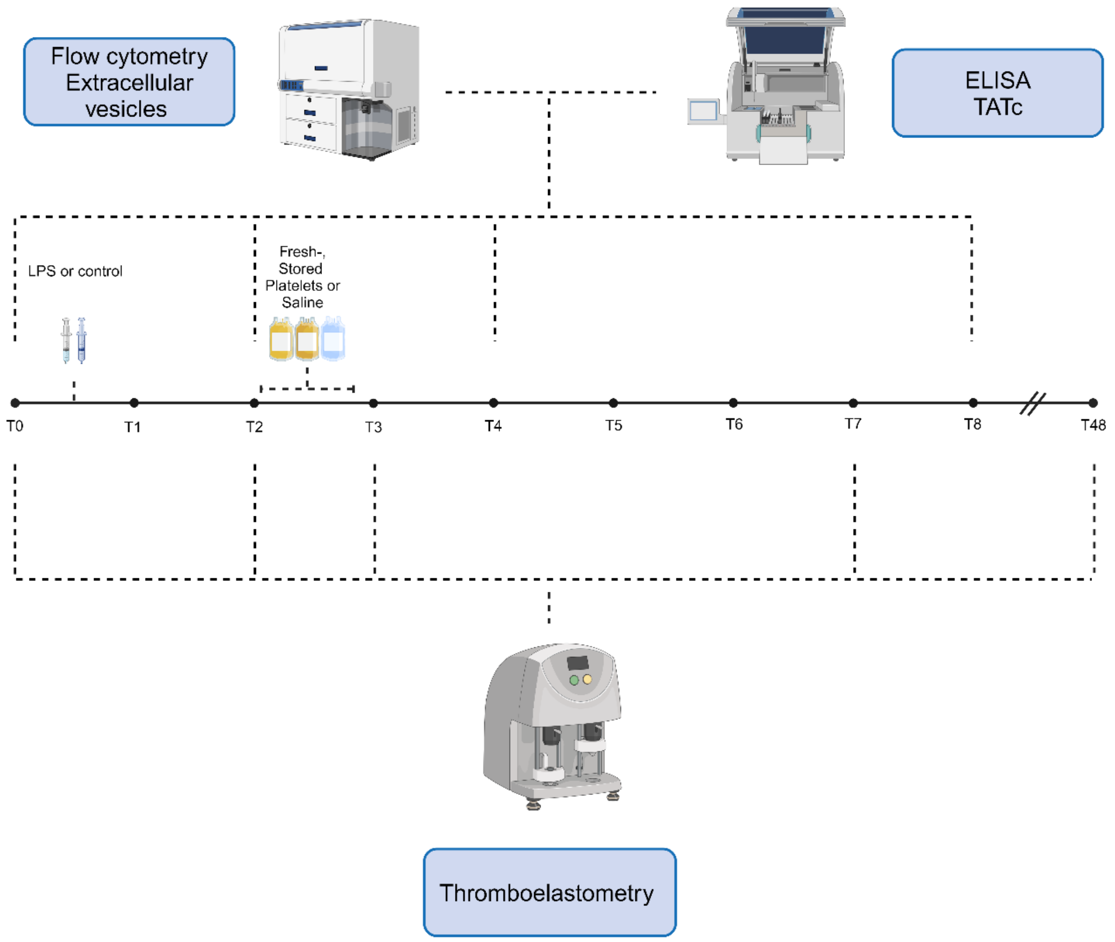

Figure S3. Change in platelet count between screening and admission. The difference in platelet count between screening and admission was calculated to evaluate the effect of donation. It is important to note that subjects receiving fresh platelets had a shorter interval (2 days) than those receiving stored platelets (7 days) between donation and transfusion. Subjects in the saline control group were split evenly, with half donating 2 days and the other half donating 7 days before the experiment.

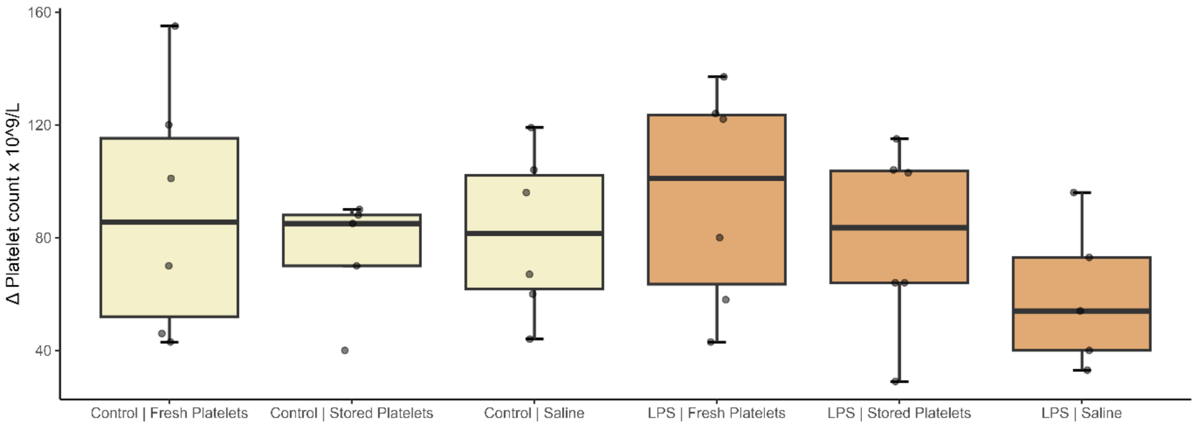

Figure S4. Platelet count increment and corrected count increment (CCI). One hour after completion of the transfusion, the platelet increment is determined by the difference in platelet count before and after the transfusion, and the corrected count increment is calculated by platelet count increment per µL × body surface area (m²) / unit content.

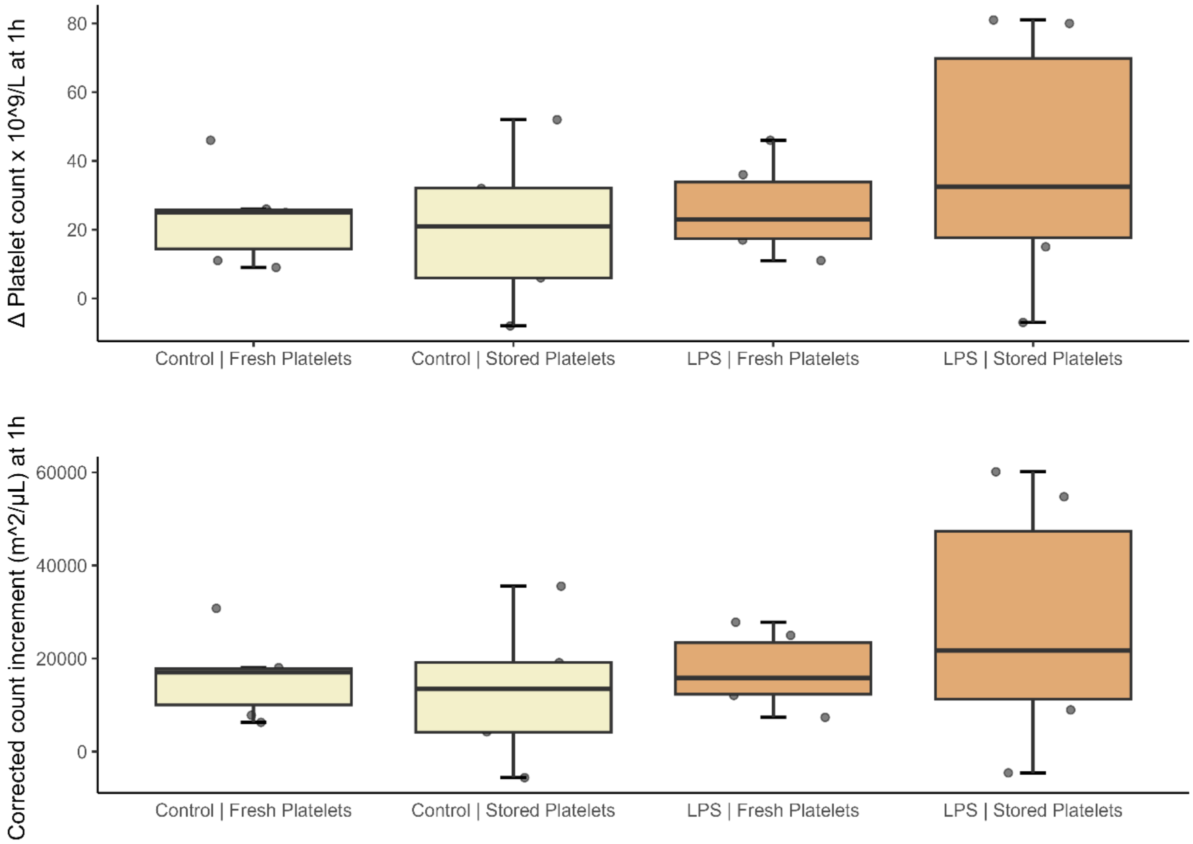

Figure S5. Circulating levels of extracellular vesicles. Extracellular vesicles were measured with flow cytometry. Data was analyzed with ANOVA repeated measurements, with main effects for intervention and time, and an interaction effect: * p<0.05 | ** p<0.01 | *** p<0.0001. Lipopolysaccharide (2ng/kg) was administered immediately after time point 0 hours and the transfusion was administered immediately after time point 2 hours.

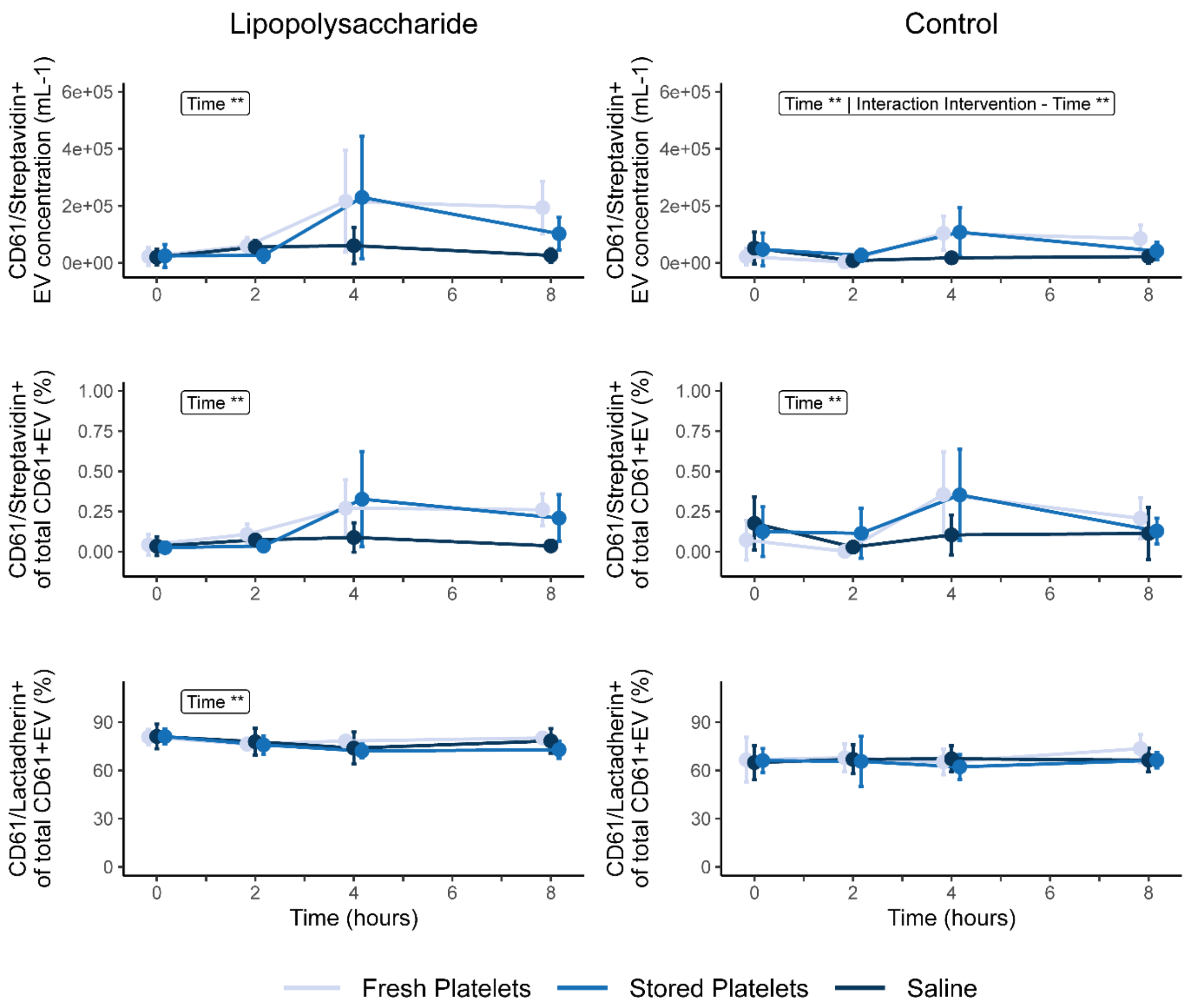

Supplement: Supplementary Figure [file mmc1.pdf]
