# Supplementary material for: Hemostatic conditions following autologous transfusion of fresh vs stored platelets in experimental endotoxemia: an open-label randomized controlled trial with healthy volunteers
Source: Res Pract Thromb Haemost. 2024 Oct 29;8(8):102612. doi: 10.1016/j.rpth.2024.102612 (PMC11616504; doi:10.1016/j.rpth.2024.102612)
Supplement: Supplementary Material [file mmc2.pdf]

MIFlowCyt-EV

Supplemental materials to:

Hemostatic Conditions Following Autologous Transfusion of Fresh versus Stored Platelets in Experimental Endotoxemia: an Open-label Randomized Controlled Trial with Healthy Volunteers

Stefan F. van Wonderen, Floor L.F. van Baarle, Anita M. Tuip-de Boer, Chantal A. Polet, Robin van Bruggen, Christie Vermeulen, Thomas R.L. Klei, Chi M. Hau, Rienk Nieuwland, Cornelis van 't Veer, Anna L. Peters, Sanne de Bruin, Alexander P.J. Vlaar, Bart J. Biemond, Marcella C.A. Müller

MIFlowCyt-EV of study “DIVA”

This document aims to provide the minimum information required to reproduce the flow cytometry experiments on extracellular vesicles (EVs) performed in the study “INOCA”. This document is based on three published standardization frameworks and guidelines [1–3].

1 Experiment overview

1.1 Contact details

1.1.1 Experiment leader

Name of organization	Amsterdam UMC location AMC
Address	Meibergdreef 9<br>PO Box 22660<br>1100 DD<br>Amsterdam<br>The Netherlands
Primary contact name	Prof. Dr. Alexander Vlaar
Primary contact e-mail address	a.p.vlaar@amsterdamumc.nl

1.1.2 Flow cytometry execution

Name of organization	Amsterdam University Medical Centers
Address	Meibergdreef 9<br>PO Box 22660<br>1100 DD<br>Amsterdam<br>The Netherlands
Contact name	C.M. Hau
Contact e-mail address	c.m.hau@amsterdamumc.nl

1.2 Purpose

The goal of this flow cytometry experiment is to investigate the impact of lipopolysaccharide injection and the storage duration of platelet concentrates on the formation of extracellular vesicles.

1.3 Keywords

Extracellular vesicles, flow cytometry, human blood plasma, LPS, platelet transfusion.

1.4 Experiment variables and model

We included 36 male volunteers aged 18-35, who were screened prior to participation and found to be healthy. Participants were not allowed to participate in any other intervention trial during the three months prior to and after the study. Subjects were allocated in 2 groups to receive a LPS (DIVA I, first 18 subjects) or a control (physiological saline: 0.9% [w/v] sodium

chloride) (DIVA II, last 18 subjects). Afterwards, subjects were randomly allocated to receive fresh autologous PC (2 days old), stored autologous PC (7 days old), or an equal volume of physiological saline.

All subjects donated 1 unit of apheresis PC (350 mL, platelet content 235 x10^9) either 2 or 7 days before the experiment, including those allocated to the saline control group, half of whom donated 2 days and half of whom donated 7 days before the experiment. PCs were collected and stored according to Dutch Blood Bank standards, and stored in 100% platelet additive solution (PAS) E. On the day of the experiment, 50mL of the PC was labeled with NHS-biotin (EZ-Link™, Thermo Fisher Scientific, Waltham, MA), according to a previously published protocol, and returned to the storage bag.

Subjects received either the autologous PC or saline, infused over 50 minutes. Overall, blood was drawn from an indwelling arterial catheter prior to PC administration, 10, 30 and 60 minutes after the transfusion and each hour thereafter, until 6 hours after the transfusion. Subjects were then discharged home and returned 48 hours after transfusion for a venous blood sample.

1.5 Experiment design and quality controls

All samples were measured using an autosampler, which facilitates subsequent measurements of samples in a 96-well plate. The entire study involved six 96-well plates that were measured within 2 months. Each well plate contained a buffer-only control, reagents in buffer controls, detergent lysis control and isotype controls. Flow rate, fluorescence, and light scattering calibrations were performed daily.

1.6 Dates

Samples were collected between April 2018 and July 2023 in the Amsterdam UMC, location AMC. Flow cytometry experiments were performed between 5 July 2022 – 8 August 2022 (DIVA I) and 14 December 2023 – 24 January 2024 (DIVA II) by the Laboratory of Experimental Clinical Chemistry (LEKC).

1.7 Data sharing

Data is available on request from a.p.vlaar@amsterdamumc.nl.

MIFlowCyt-EV of study “DIVA I”

2 Sample details

2.1 Sample description

2.1.1 Sample source description

We enrolled 18 healthy male volunteers aged 18–35 in the first part of study to receive LPS prior transfusion. If any volunteers withdrew from the study between the screening visit and subsequent study visits, they were replaced. Volunteers underwent screening involving medical history review, physical examination, blood and urine tests, electrocardiogram (ECG), chest X-ray, spirometry, and measurement of lung diffusion capacity for carbon monoxide (DLCO). Previous participation in a trial involving lipopolysaccharide (LPS) was not permitted, and volunteers were ineligible if they had participated in another intervention trial within 3 months prior to inclusion or during the study period.

2.1.2 Sample description

Arterial blood.

2.2 Sample collection

Blood collected in 3 mL citrate tube (BD Vacutainer, New Jersey, US) via an arterial catheter without tourniquet. The first 2 mL of collected blood were discarded to avoid that activation of endothelial cells and platelets which potentially affect the measurement results.

2.3 Sample storage

Within 15 minutes from blood collection, plasma was prepared by double centrifugation using a Rotina 380 R equipped with a swing-out rotor and a radius of 155 mm (Hettich Zentrifugen, Tuttlingen, Germany). The centrifugation parameters were: 2,500 g, 15 minutes, 20˚C, acceleration speed 9, deceleration speed 1. The first centrifugation step was done with 3 mL whole blood collection tubes. Supernatant was collected 10 mm above the buffy coat. The second centrifugation step was done with 1.5 mL plasma in 10 mL polypropylene tubes (Sarstedt, Nümbrecht, Germany). Supernatant was collected 10 mm from the bottom of the tube, transferred into 1.5 mL tubes (Greiner-one, Kremsmünster, Austria), mixed by pipetting, aliquoted in volumes of 100 µL, transferred to 1.5 mL micro tube (Sarstedt, Nümbrecht, Germany), snap frozen in liquid nitrogen and stored in -80˚C.

2.4 Sample characteristics

Frozen human plasma samples are expected to contain the following particles: erythrocyte ghosts, EVs, lipoproteins, platelets, precipitated salt crystals, proteins, and complexes of the aforementioned particles.

3 Flow cytometry

3.1 Experimental design

The aim of flow cytometry (A60-Micro, Apogee Flow Systems, Hemel Hempstead, UK) experiments was to measure the concentrations of extracellular vesicles (EVs) released from biotin-labeled platelets (steptavidin$^+$ / CD61$^+$)] in platelet-depleted plasma (PPP) samples to investigate whether longer stored platelets release more EVs than shorter stored platelets. We hypothesized that 7-day stored platelets would release more EVs than 2-day stored platelets.

All samples were measured using an autosampler, which facilitates subsequent measurements of samples in a 96-well plate. For this study, samples were measured on 6 different days (05 July 2022 – 08 August 2022). Each day a buffer-only control was measured as well as antibody in buffer controls and isotype controls corresponding to the labels in the well plate. Flow rate and scatter calibrations were performed daily. Fluorescence calibration was performed once during this time period. To automatically determine optimal sample dilutions, apply calibrations, determine and apply gates, generate reports with scatter plots and generate data summaries, we used custom-build software (MATLAB R2018b, Mathworks, Natick, MA, USA).

3.2 Sample dilutions

To avoid swarm detection in typical PPP samples, we measured serial dilutions of PPP pooled from 10 healthy male and 10 healthy female donors. Figure 1A and 1B show the measured concentration and median side scattering signals versus dilution of pooled PPP, respectively. For measured concentrations ≤ $1.5 \cdot 10^8$ mL^{-1} and count rates ≤ $7.5 \cdot 10^3$ events per second, the concentration decreases linearly with the dilution and the median side scattering signals are similar, confirming absence of swarm detection.

Based on the result in Figure 1, and to be on the safe side, we aimed for count rates ≤ $1.0 \cdot 10^4$ events per second for all measurements. Pre-staining, PPP samples were diluted 10 – 200 times, followed by an additional 11.375-fold dilution post-staining, resulting in count rates between 354 – 9,155 events per second. More research is required, however, to confirm that the count rate can be used as a benchmark to avoid swarm detection for a given sample type.

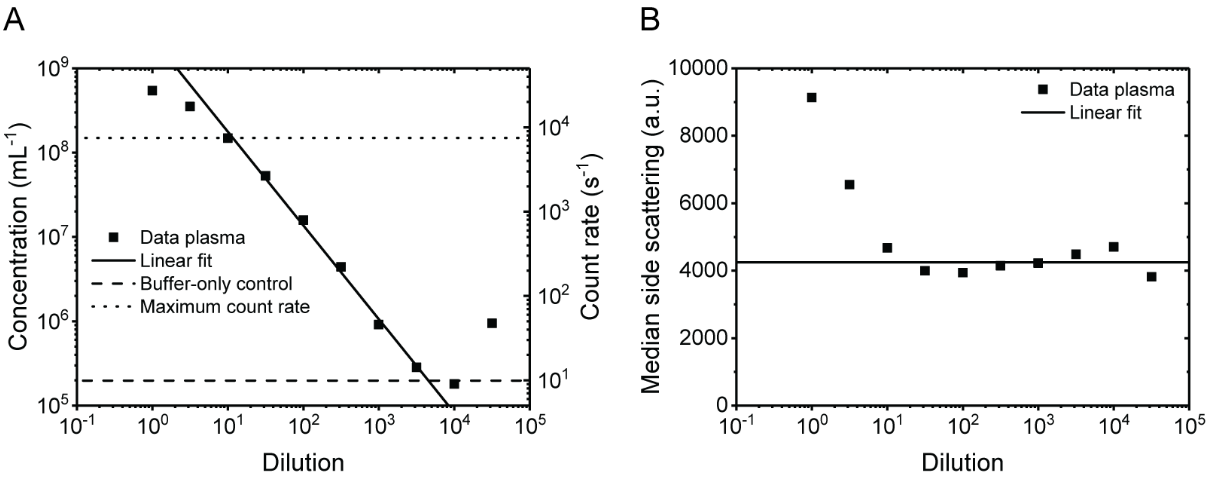

Figure 1. Estimation of the minimum dilution and maximum count rate to avoid swarm detection for particles in pooled platelet poor plasma (PPP). (A) Particle concentration and count rate versus dilution measured (symbols) with the A60-Micro in pooled PPP and fitted with a linear function (solid line; slope -1.11, intercept 9.36, R^2=0.996) based on datapoints 3 to 8. As a reference, the concentration and count rate of the buffer-only control (dashed line) is shown. (B) Median side scattering versus dilution measured (symbols) with the A60-Micro in pooled PPP and fitted with a linear function (solid line) based on datapoints 3 to 10. For concentrations ≤ $1.5 \cdot 10^8$ mL^{-1} and count rates ≤ $7.5 \cdot 10^3$ events per second (dotted line in panel A), the measured concentration scales linearly with the reciprocal dilution and the median side scatter signals are similar.

3.3 EV staining

EVs were stained with antibodies. Prior to staining, antibodies were diluted in DPBS and centrifuged at 18,890 g for 5 min to remove aggregates. Table 1 shows an overview of the used reagents and antibody concentrations during staining. To stain, 20 µL of pre-diluted PPP was incubated with 2.5 µL of antibodies or isotype controls and kept in the dark for 2 h at room temperature. Post-staining, samples were diluted 11.375-fold in 200 µL of DPBS to decrease background fluorescence from unbound reagents.

3.4 Buffer-only control

Each measurement day at least 1 well with DPBS was measured with the same flow cytometer and acquisition settings as the samples. The mean count rate was 99 events per second, which is substantially lower than the count rates obtained for PPP samples (354 – 9,155 events per second).

3.5 Buffer with reagents control

Each 96-wellplate contained a buffer with reagent control for each reagent (Table 1), which was measured with the same flow cytometer and acquisition settings as the samples. For all reagents, the mean count rate was between 37 - 111 events per second, which is in the same range as the buffer-only control.

3.6 Unstained controls

Unstained controls were measured with the same flow cytometer and acquisition settings as the stained samples, resulting in 348 – 8,972 events per second for PPP samples.

3.7 Isotype controls

Table 1 shows an overview of the used isotype controls, which were added to a selection of samples. For 126 samples, we obtained an average of 4 IgG1-APC+ events, 94 IgG1-BV421+ and 160 IgG1-PE+ events with a diameter ≤1,000 nm per measurement. For comparison, on average 1,578 CD45-APC+, 108 Streptavidin-APC+, 6,841 CD61-BV421+, 1,464 CD235a-PE+ and 15,423 Lactadherin-FITC+ events with a diameter ≤1,000 nm were obtained in the experiments using PPP samples.

3.8 Trigger channel and threshold

Based on the buffer-only control (99 events s^{-1}), the acquisition software was set up to trigger at 24 arbitrary units (a.u.) SSC, which is equivalent to an SSC cross section of 10 nm^2 (Rosetta Calibration, v1.29, Exometry, Amsterdam, The Netherlands).

3.9 Flow rate quantification

The A60-Micro is equipped with a syringe pump with volumetric control, which we checked on a daily basis using ApoCal Mix (Apogee calibration beads, Apogee Flow Systems, Hemel Hempstead, UK). Here, we assumed that the flow rate is equal to the adjusted flow rate of 3.01 µL/min for all measurements.

3.10 Fluorescence calibration

Calibration of the fluorescence detectors from arbitrary units (a.u.) to molecules of equivalent soluble fluorochrome (MESF) was accomplished using 2 µm APC Quantitative Beads (2321-175, BD), 3 µm BV421 Quantitative Beads (2337-25, BD), 2 µm FITC Quantitative Beads (2364-85, BD) and 2 µm PE Quantitative Beads (2364-89, BD)

Calibration of the APC, BV421, FITC and PE detector was performed once in the time period of these experiments. Calibration correction for FITC detector was performed once during data analysing. For each measurement, we added fluorescent intensities in MESF to the flow cytometry data files by custom-build software (MATLAB R2018a) using the following equation:

$$\mathrm{I(MESF)} = 10^{a\cdot\log_{10}\mathrm{I(a.u.)}+b} \quad \text{Equation S1}$$

where I is the fluorescence intensity, and *a* and *b* are the slope and the intercept of the linear fits, respectively, see Table 2.

3.11 Light scatter calibration

We used Rosetta Calibration to relate scatter measured by FSC or SSC to the scattering cross section and diameter of EVs. Figure 2 shows print screens of the scatter calibrations. We modelled EVs as core-shell particles with a core refractive index of 1.38, a shell refractive index of 1.48, and a shell thickness of 6 nm. For each measurement, we added the

FSC and SSC scattering cross sections and EV diameters to the flow cytometry datafiles by custom-build software (MATLAB R2018a).

3.12 MIFlowCyt checklist

The MIFlowCyt checklist is added to Table 3.

3.13 EV number concentration

The concentrations reported in the manuscript describe the number of particles (1) that exceeded the SSC threshold, corresponding to a side scattering cross section of 10 nm^2, (2) that were collected during time intervals, for which the count rate did not deviated more than 750 events/s of the median count rate, (3) with a diameter <1,000 nm as measured by SSC after light scatter calibration (section 1.11) and (4) are positive for APC, BV421, PE or FITC, per mL of PPP.

Table 1: Overview of staining reagents. Characteristics being measured, analyte, analyte detector, reporter, isotype, clone, concentration, manufacturer, catalog number and lot number of used staining reagents. The antibody concentration during measurements was 11.25-fold lower than the antibody concentration during staining.

Characteristic measured	Analyte	Analyte detector	Reporter	Isotype	Clone	Concentration during staining (µg mL^{-1})	Manufacturer	Catalog number	Lot number
Integrin	Human CD61	Anti-human CD61 antibody	BV421	IgG1	VI-PL2	3.125	BD Bioscience	744381	191784
Leukocyte common antigen	CD45	Anti-human CD45 antibody	APC	IgG1	HI30	4.5	BioLegend	304037	B272158
Glyco-protein	CD235a	Anti-human CD235a antibody	PE	IgG1	JC159	12.5	Dako Agilent	R7078	41236187
Second-step reagent, binding to biotinylated primary antibody	Streptavidin	Streptavidin	APC	n.a.	n.a.	5.0	BD Bioscience	554067	1286144
Phosphatidyl serin	Bovin Lactadherin	Lactadherin	FITC	n.a.	n.a.	20.8	Prolytix	BLAC-FITC	MM0301
Affinity for Fc receptor	Fc receptor	IgG1	BV421	n.a.	X40	3.125	BD Bioscience	562438	8242928
Affinity for Fc receptor	Fc receptor	IgG1	APC	n.a.	MOPC-21	4.5	BD Bioscience	554681	8261691
Affinity for Fc receptor	Fc receptor	IgG1	PE	n.a.	X40	12.5	BD Bioscience	345816	9309643

APC: allophycocyanin; BV421: brilliant-violet 421; CD: cluster of differentiation; FITC: fluorescein isothiocyanate; IgG: immunoglobulin G; PE: phycoerythrin.

Table 2: Overview of fluorescence calibrations.

	Calibration date	Slope	Intercept	R^2
APC	2022-03-01	1.1936	-2.4899	0.9949
BV421	2022-03-03	1.0726	-1.7499	0.9996
FITC	2022-03-01<br>Calibration correction	1.1823<br>1.1823	-2.1922<br>-1.9786	0.9993
PE	2022-03-29	1.2538	-2.757	0.9916

APC: allophycocyanin; BV421: brilliant-violet 421; FITC: fluorescein isothiocyanate; PE: phycoerythrin.

Table 3. MIFlowCyt checklist.

Requirement	Please Include Requested Information
1.1. Purpose	To investigate whether longer stored (7 days) platelets release more extracellular vesicles than fresh (2 days stored) platelets after transfusion during experimental endotoxemia in healthy adults.
1.2. Keywords	Endotoxemia; Platelet Transfusion; Platelet Storage Lesion
1.3. Experiment variables	Healthy volunteers were randomized to receive either fresh (2-day stored) or stored (7-day stored) autologous platelet concentrate, or an equal volume (300cc) of normal saline, two hours after a first hit of 2ng/kg lipopolysaccharide.
1.4. Organization name and address	Amsterdam University Medical Centers<br>Location Academic Medical Centre<br>Meibergdreef 9<br>1105 AZ Amsterdam<br>The Netherlands
1.6. Date or time period of experiment	05 July 2022 – 8 August 2022
1.7. Conclusions	There is no difference in EVs released from fresh and stored platelets.
1.8. Quality control measures	All samples were measured using an autosampler, which facilitates subsequent measurements of samples in a 96-well plate. Each well plate contained buffer-only controls (section S1.4), antibody in buffer controls (section S1.5), unstained controls (section S1.6) and isotype controls (section S1.7). The flow rate is calibrated by ApoCal Mix (Apogee calibration beads, Flow Systems, Hemel Hempstead, UK; section S1.9). Fluorescence detectors were calibrated (section S1.10) with 2 µm APC Quantitative Beads (2321-175, BD), 3 µm BV421 Quantitative Beads (2337-25, BD), 2 µm FITC Quantitative Beads (2364-85, BD) and 2 µm PE Quantitative Beads (2364-89, BD). FSC and SSC were calibrated with Rosetta Calibration (v1.27, section S1.11).
1.9 Other relevant experiment information	Samples were measured on 6 different days, in a time period of 2 months.
2.1.1.1. Sample description	The plasma samples analysed in this study are platelets poor plasma that was obtained prior to LPS infusion, prior to transfusion and 1 and 5 hours after transfusion.
2.1.1.2. Biological sample source description	Blood samples were collected in plastic vacuum tubes 3 mL citrate; final concentration 0.109 mol/L (BD Vacutainer®, USA), via a radial artery catheter, using a needleless blood sampling system (VAMP system, Edwards Lifesciences, Irvine, CA, USA). Platelet-depleted plasma was prepared by double centrifugation using a Rotina 380 R equipped with a swing-out rotor and a radius of 155 mm (Hettich Zentrifugen, Tuttlingen, Germany). The

	centrifuged parameters were: 2,500g, 15 minutes, 20˚C, no brake. For freeze-storage, samples were transferred to 1.5 mL micro tubes (Sarstedt AG & Co., Germany), snap-frozen in liquid nitrogen and stored in -80˚C. Before staining, samples were thawed for 1 minute at 37°C.
2.1.1.3. Biological sample source organism description	Healthy human male volunteers
2.2 Sample characteristics	Platelet-depleted plasma (PPP) is expected to contain EVs, lipoproteins and proteins. There might be some residual platelets present.
2.3. Sample treatment description	Samples were brought to the lab by manual transport in vertical position. Plasma was prepared by double centrifugation and samples were stained. Please see section S1.3 for staining procedure.
2.4. Fluorescence reagent(s) description	Please see Table 1.
3.1. Instrument manufacturer	Apogee, Hemel Hempstead, UK
3.2. Instrument model	A60-Micro
3.3. Instrument configuration and settings	Samples were analysed for 2 minutes at a flow rate of 3.01 µL/min on an A60-Micro, equipped with a 405 nm laser (100 mW), 488 nm laser (150 mW) and 638 nm laser (150 mW). The trigger threshold was set at SSC 24 a.u. corresponding to an SSC cross section of 10 nm^2 (Rosetta Calibration).<br>For FSC and SSC, the PMT voltages were 470 V and 375 V, respectively. For all detectors, the peak height was analysed. APC signals were collected with the 638-D Red(Peak) detector (long pass 650 nm filter, PMT voltage 480 V). BV421 signals were collected with the 405-Blue(Peak) detector (445/50 nm band pass filter, PMT voltage 460 V). FITC signals were collected with the 488-Green(Peak) detector (525/50 nm band pass filter, PMT voltage 560 V). PE signals were collected with the 488-Orange(Peak) detector (575/30 nm band pass filter, PMT voltage 450 V).
4.1. List-mode data files	Data is available on request from a.p.vlaar@amsterdamumc.nl.
4.2. Compensation description	No compensation was required because no fluorophore combinations were used that have overlapping emission spectra.
4.3. Data transformation details	Fluorescence detectors were calibrated (section S1.10) with 2 µm APC Quantitative Beads (2321-175, BD), 3 µm BV421 Quantitative Beads (2337-25, BD), 2 µm FITC Quantitative Beads (2364-85, BD) and 2 µm PE Quantitative Beads (2364-89, BD). FSC and SSC were calibrated with Rosetta Calibration (v1.27, section S1.11). The concentrations reported in the manuscript describe the number of particles that fulfil the gating criteria per mL.

4.4.1. Gate description	To automatically apply gates, generate pdf reports with scatter plots, and summarize the data in a table, custom-build software (MATLAB R2018b) was used. Please find below a description of the gates. First, events that were collected during seconds for which the count rate deviated less than 750 events/seconds from the median count rate were included. Second, events with a diameter <1,000 nm as measured by SSC after light scatter calibration (section S1.11) were included. Third, events positive for either APC, BV421, FITC or PE were included.
4.4.2. Gate statistics	The number of positive events was corrected for flow rate, measurement time and dilutions performed during sample preparation.
4.4.3. Gate boundaries	The lower boundaries of the fluorescent gates were automatically determined (MATLAB R2018b). For platelet-depleted plasma samples they were 86 MESF for CD61-BV421, 40 MESF for CD235a-PE, 46 MESF for CD45-APC, 65 MESF for Streptavidin-APC and 212 MESF for Lactadherin-FITC.

a.u.: arbitrary units; EVs: extracellular vesicles; FSC: forward scattering; PPP: platelet depleted plasma; SSC: side scattering.

MIFlowCyt-EV of study "*DIVA II study*"

4 Sample details

4.1 Sample description

4.1.1 Sample source description

We enrolled 18 healthy male volunteers aged 18–35 in the second part of study to receive saline as control (instead of LPS) prior transfusion. If any volunteers withdrew from the study between the screening visit and subsequent study visits, they were replaced. Volunteers underwent screening involving medical history review, physical examination, blood and urine tests, electrocardiogram (ECG), chest X-ray, spirometry, and measurement of lung diffusion capacity for carbon monoxide (DLCO). Previous participation in a trial involving lipopolysaccharide (LPS) was not permitted, and volunteers were ineligible if they had participated in another intervention trial within 3 months prior to inclusion or during the study period.

4.1.2 Sample description

Arterial blood.

4.2 Sample collection

Blood collected in 3 mL citrate tube (BD Vacutainer, New Jersey, US) via an arterial catheter without tourniquet. The first 2 mL of collected blood were discarded to avoid that activation of endothelial cells and platelets which potentially affect the measurement results.

4.3 Sample storage

Within 15 minutes from blood collection, plasma was prepared by double centrifugation using a Rotina 380 R equipped with a swing-out rotor and a radius of 155 mm (Hettich Zentrifugen, Tuttlingen, Germany). The centrifugation parameters were: 2,500 g, 15 minutes, 20˚C, acceleration speed 9, deceleration speed 1. The first centrifugation step was done with 3 mL whole blood collection tubes. Supernatant was collected 10 mm above the buffy coat. The second centrifugation step was done with 1.5 mL plasma in 10 mL polypropylene tubes (Sarstedt, Nümbrecht, Germany). Supernatant was collected 10 mm from the bottom of the tube, transferred into 1.5 mL tubes (Greiner-one, Kremsmünster, Austria), mixed by pipetting, aliquoted in volumes of 100 μL, transferred to 1.5 mL micro tube (Sarstedt, Nümbrecht, Germany), snap frozen in liquid nitrogen and stored in -80˚C.

4.4 Sample characteristics

Frozen human plasma samples are expected to contain the following particles: erythrocyte ghosts, EVs, lipoproteins, platelets, precipitated salt crystals, proteins, and complexes of the aforementioned particles.

4.5 Sample dilution

As the concentration of particles in plasma differs $>10^2$-fold between donors, samples require different dilutions to (1) avoid swarm detection and (2) detect a statistically significant number of events within a measurement time of a few minutes. The optimal dilution factor is the minimum dilution factor that is required to prevent swarm detection. For the flow cytometer and settings used, the optimal dilution factor for plasma is $\geq 1.1 \cdot 10^2$ -fold and should result in a count rate $<1.1 \cdot 10^4$ events$\cdot s^{-1}$ [4].

To find the dilution resulting in a count rate $<1.1 \cdot 10^4$ events$\cdot s^{-1}$, we diluted each sample 200- fold and 2,000-fold in Dulbecco′s phosphate buffered saline (DPBS) and measured the total concentration of particles for 30 s without staining. For all experiments, DPBS (Corning, New York, US) was used. By diluting each sample 200-fold and 2,000-fold, all samples had a count rate $<1.1 \cdot 10^4$ events$\cdot s^{-1}$. Figure 2A shows a distribution of the measured total particle concentrations of all samples in the study. Taking into account the measured concentration and flow rate, we calculated the minimum dilution factor required before staining (section 4.6) to achieve a count rate $<1.1 \cdot 10^4$ events$\cdot s^{-1}$ after staining. The staining procedure adds an extra dilution factor of 11.1-fold to the overall dilution. To simplify the pipetting procedures, samples were divided into 6 categories of pre-staining dilution factors: 10-fold, 16-fold, 25-fold, 40-fold, 65-fold, 100-fold, 160-fold and 260-fold. Figure 2B shows a distribution of the applied pre-staining dilution factors of all samples in the study.

4.6 Sample staining

Table shows an overview of the antibodies that were used to stain EVs in plasma. Prior to staining, the antibodies were diluted in DPBS. For each antibody the optimal dilution factor was determined by titration (Table 4). To remove aggregates, diluted antibodies were centrifuged at 18,890 g for 5 min at 20 °C. The supernatant minus 10 μL of the starting volume was collected and used for staining. Each sample was triplicate stained with CD45-APC (allophycocyanin), CD61-BV421 (brilliant violet 421) and CD235a-PE (phycoerythrin), and with Streptavidin-APC (allophycocyanin), CD61-BV421 (brilliant violet 421) and Lactadherin-FITC (fluorescein isothiocyanate). To stain, 20 μL of pre-staining diluted (*Figure* 2B) plasma was incubated with 2.5 μL of each antibody or isotype controls and kept in the dark for 2 h at room temperature. After the incubation, samples were diluted in 200 μL DPBS to decrease background fluorescence from unbound reagents.

4.7 Fluorescence reagents

Characteristic measured	Analyte	Analyte detector	Reporter	Isotype	Clone	Concentration (μg mL^{-1})	Manufacturer	Catalog number	Lot number	Dilution factor
Integrin	Human CD61	Anti-human CD61 antibody	BV421	IgG1	VI-PL2	200	BD Bioscience	744381	3101001	64x
Leukocyte common antigen	Human CD45	Anti-human CD45 antibody	APC	IgG1	HI30	9	Biolegend	304037	B311564	2x
Erythrocyte antigen	CD235a	Anti-human CD235a antibody	PE	IgG1	JC159	100	Dako	R7078	41236187	8x
Second-step reagent for indirect staining	Streptavidin	Biotinylated primaire antibody	APC	n/a	n/a	200	BD Bioscience	554067	1286144	40x
Phosphatidyl serin	Bovine Lactadherin	Lactadherin	FITC	n/a	n/a	83	Prolytix	LACT-FITC	NN0427	4x
Affinity for Fc receptor	Fc receptor	IgG1 κ	APC	n/a	MOPC-21	200	BD Bioscience	554681	8261691	44x
	Fc receptor	IgG1	BV421	n/a	X40	200	BD Bioscience	562438	8242926	64x
	Fc receptor	IgG1	PE	n/a	X40	50	BD Bioscience	345816	9309643	8x

Table 4. Overview of staining reagents. Characteristics being measured, analyte, analyte detector, reporter, isotype, clone, concentration during staining, manufacturer, catalog number and lot number of used staining reagents. The concentration of staining reagents during measurements was 11.1-fold lower than the concentration during staining. APC: allophycocyanin; BV421: Brilliant Violet 421; CD: cluster of differentiation; FITC: fluorescein isothiocyanate; IgG: immunoglobulin G; PE: phycoerythrin; BD: Becton Dickinson.

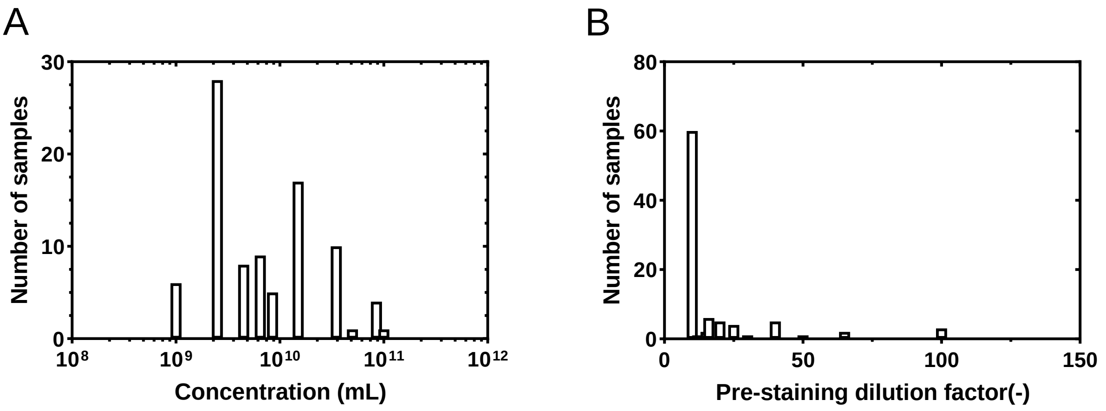

Figure 2. (A) Distribution of the total concentration of particles exceeding the trigger threshold for all samples in the study. (B) Distribution of the applied pre-staining dilution factors for all samples in the study.

5 Flow cytometer

5.1 Model and manufacturer

A60-Micro, Apogee Flow Systems, Hemel Hempstead, UK. The flow cytometer has not been altered. All components are original and came with the flow cytometer.

5.2 Configuration and settings

5.2.1 Flow rate and acquisition time

The flow cytometer is equipped with a syringe pump with volumetric control. Samples were analysed for 120s or 240 s at a flow rate of 3.01 μL/min.

5.2.2 Light sources

The flow cytometer has three lasers that illuminate a fixed-alignment cuvette flow cell. The laser powers were 100 mW, 150 mW and 150 mW for the 405-nm, 488-nm, and 638-nm laser, respectively.

5.2.3 Detectors

Table 5 shows an overview of the detectors used in this study.

Detector name	Detected property	Voltage (V)	Spectral filter bandwidth (nm)
405-SALS	Forward scattered light	470	
405-LALS	Side scattered light	367	
405-Blue	BV421 fluorescence	460	445/50
488-Orange	PE fluorescence	450	575/30
488-Green	FITC fluorescence	560	525/50
638-D Red	APC fluorescence	480	>650

Table 5. Detector name, detected property, voltage and spectral filter bandwidth of the detectors used in this study. APC: allophycocyanin; BV421: Brilliant Violet 421; FITC:

fluorescein isothiocyanate; PE: phycoerythrin; SALS: small angle light scattering; LALS: large angle light scattering.

5.2.4 Trigger detector and threshold

Based on the buffer-only control, a trigger threshold of 16 arbitrary units was applied to the side scattering detector. As (1) the A60-Micro applies the trigger threshold analogically, thus before digitalization of the signal, and (2) the arbitrary unit channel numbers differ between the data acquisition software and the flow cytometry datafiles, the trigger threshold was expressed in standard units by taking the mode of the effective side scattering cross section distribution and optical diameter distribution (section 7.3.3.1) of a plasma sample. Plasma samples have a size distribution with a peak far below the detection limit of the A60-Micro. The modes of the effective side scattering cross section distribution and optical diameter distribution therefore represent the trigger threshold. The trigger threshold is equivalent to an effective side scattering cross section of 10 nm^2 and an optical diameter of 165 nm for EVs[1].

6 Assay controls

Assay controls recommended by the MIFlowCyt-EV framework were performed to confirm that signals originate from EVs. Fluorescence-minus-one and single-stained controls were not performed due to thorough experience with the used antibody panels and because the emission spectra of the used fluorophores do not have spectral overlap. Procedural controls were not performed because no methods to isolate EVs were applied after staining. Serial dilution control were performed on the A60-Micro for six representative plasma samples and results were published [4]. Section 4.5 explains how swarm detection was prevented.

6.1 Buffer-only controls

Each 96-well plate contained at least 1 well with DPBS, which was measured with the same flow cytometer and acquisition settings as all other samples. The median count rate for all DPBS measurements was 39 events s^{-1}, which is lower than the target count rate ($7.0 \cdot 10^3$ events s^{-1}) for events in stained plasma samples.

6.2 Buffer with reagents controls

Each 96-wellplate contained a buffer with reagent control for each reagent (Table 4), which was measured with the same flow cytometer and acquisition settings as all other samples. Table 6 shows a summary of the results of the buffer with reagents controls.

Reagent	Mean number of fluorescence positive events in buffer (120 s^{-1})	Mean number of fluorescence positive events in stained samples (120 s^{-1})	Mean number of fluorescence positive events in buffer / stained samples (-)

[1] EVs are modelled as core-shell particles with a core refractive index of 1.38, a shell refractive index of 1.48, and a shell thickness of 6 nm.

CD61-BV421	23	1575	0.0143
CD45-APC	45	435	0.1031
CD235a-PE	13	1296	0.0102
Streptavidin-APC	0	14	0.0237
Lactadherin-FITC	74	2915	0.0252

Table 6. Results of the buffer with reagents controls. APC: allophycocyanin; BV421: Brilliant Violet 421; CD: cluster of differentiation; FITC: fluorescein isothiocyanate; PE: phycoerythrin.

6.3 Unstained controls

Unstained controls were measured with a 200-fold and 2,000-fold dilution factor, which differs from the dilution factor with which the stained plasma samples were measured. Unstained controls were not considered in this analysis.

6.4 Isotype controls

Each 96-wellplate contained an arbitrary plasma sample stained with IgG1, which was measured with the same flow cytometer and acquisition settings as all other samples. Table 7 shows a summary of the results of the isotype controls.

Sample	Mean number of fluorescence positive events in isotype control (120 s^{-1})	Mean number of fluorescence positive events in stained samples (120 s^{-1})	Mean number of fluorescence positive events in isotype control / stained samples (-)
CD61-BV421	34	1575	0.0215
CD45-APC	0	435	0.0007
CD235a-PE	160	1296	0.1237

Table 7. Results of the isotype controls. APC: allophycocyanin; BV421: Brilliant Violet 421; CD: cluster of differentiation; PE: phycoerythrin.

6.5 Detergent treatment controls

One 96-wellplate contained an arbitrary plasma sample to which 10% NP-40 (Merck Life Science, The Netherlands) was added. After vortexing for 5 seconds, the sample was measured with the same flow cytometer and acquisition settings as all other samples. Table 8 shows a summary of the results of the detergent treatment controls.

Reagent	Number of fluorescence positive events after detergent treatment (120 s^{-1})	Number of fluorescence positive events in stained samples (120 s^{-1})	Number of fluorescence positive events after detergent treatment / stained samples (-)

CD61-BV421	150	982	0.1527
CD45-APC	25	393	0.0636
CD235a-PE	50	366	0.1366

Table 8. Results of the detergent treatment controls. APC: allophycocyanin; BV421: Briiliant Violet 421; CD: cluster of differentiation;PE: phycoerythrin.

7 Data analyses

To automatically determine optimal samples dilutions, apply calibrations, determine and apply gates, generate reports with scatter plots and generate data summaries, we developed and applied custom-build software (MATLAB R2020b, Mathworks, USA).

7.1 Data sharing

Data are available via: a.p.vlaar@amsterdamumc.nl

7.2 Compensation details

No compensation was applied because no fluorophore combinations were used that have overlapping emission spectra.

7.3 Calibrations

7.3.1 Flow rate

At the start of each measurement day, we applied the automated quality control system ApoCal (#1524, Apogee Flow Systems), which checks whether the flow rate is within 20% of the adjusted flow rate of 3.01 $\mu L \cdot min^{-1}$. For all days, the flow cytometer passed this quality control check.

7.3.2 Fluorescence calibration

Calibration of the fluorescence detectors from arbitrary units (a.u.) to molecules of equivalent soluble fluorochrome (MESF) was accomplished using 2 μm APC quantification beads (lot 2364-19, custom-order, Becton Dickinson Biosciences), 3 μm BV421 Quantitative Beads (lot 2337-25, custom-order, Becton Dickinson Biosciences), 2 μm FITC Quantitative Beads (lot 2364-85, custom-order, Becton Dickinson Biosciences) and 2 μm PE Quantification beads (lot 2364-89, custom-order, Becton Dickinson Biosciences). Figure 3A and B show the 10-base logarithm of the MESF intensities for the MESF beads versus the 10-base logarithm of the measured median fluorescence intensity of each bead population. The data are fitted with a linear function. These fluorescence calibrations were used to assign MESF values for APC, BV421, FITC and PE to rainbow beads (SPHERO^{TM} Rainbow calibration particles, 8 peaks, 3.0-3.4 μm, lot EAP01, Spherotech). In turn, the rainbow beads, which are hard dyed beads with long-term stability, were used to apply fluorescence calibrations on a daily basis. Figure 3C and D show the 10-base logarithm of the assigned MESF intensities for rainbow beads versus the 10-base logarithm of the measured median fluorescence intensity of each bead population. The data are fitted with a linear function. For each measured plasma

sample, we added fluorescent intensities in MESF units to the flow cytometry data files using following equation:

$\mathrm{I(MESF)} = 10^{a\cdot\log_{10}\mathrm{I(a.u.)}+b}$	Equation 1

where I is the fluorescence intensity, and *a* and *b* are the slope and the intercept of the linear fits in Figure 3.

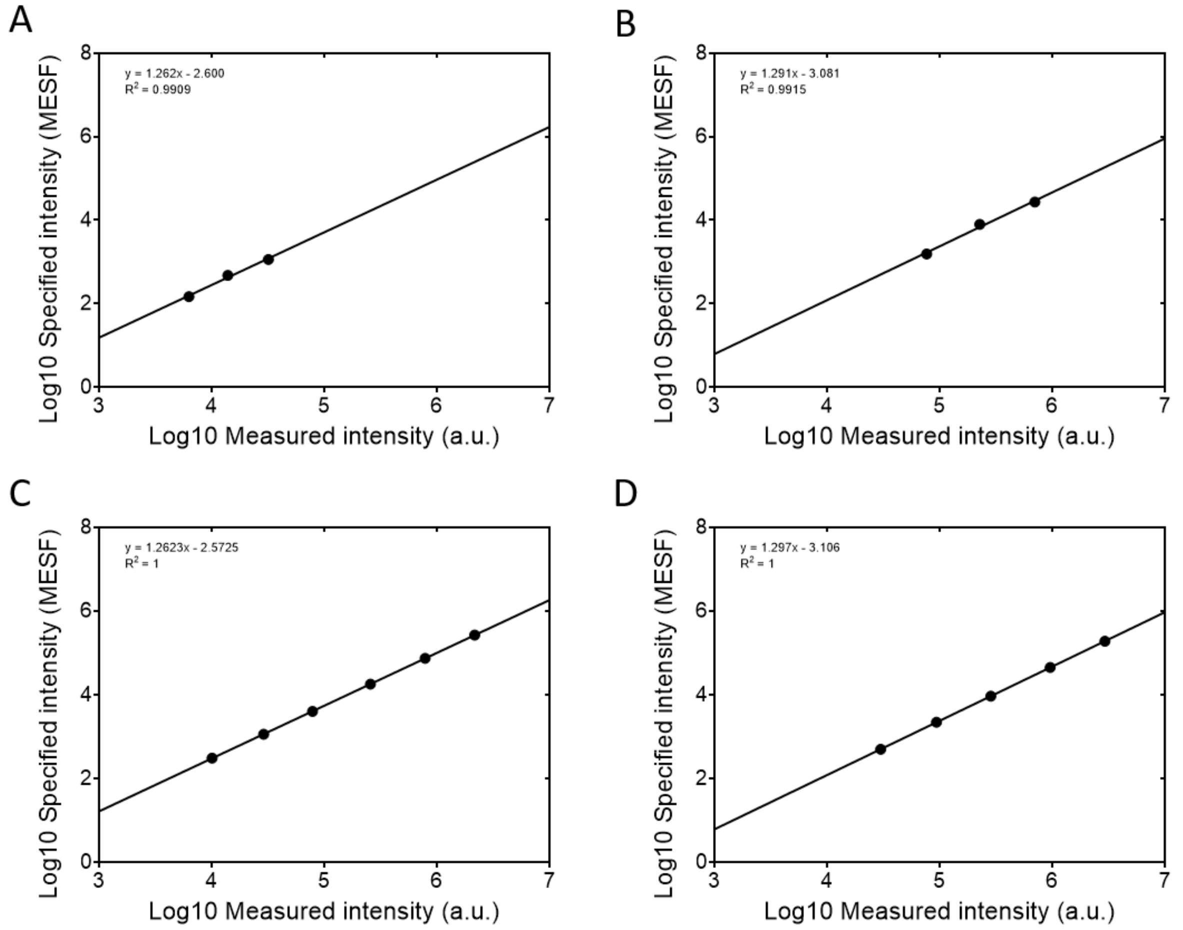

Figure 3. Calibration of the fluorescence detectors from arbitrary units (a.u.) to molecules of equivalent soluble fluorochrome (MESF). Logarithmic MESF versus logarithmic median fluorescence intensity for (A) phycoerythrin (PE), and (B) allophycocyanin (APC). Furthermore, the 10-base logarithm of the assigned MESF intensities for rainbow beads versus 10-base logarithm of the measured median fluorescence intensity of each bead population are shown for PE (C), and APC (D). Data (symbols) are fitted with a linear function (line).

7.3.3 Light scattering calibration

7.3.3.1 Rosetta Calibration

Rosetta Calibration (v2.05, Exometry, The Netherlands) was used to relate the forward and side scattering intensities measured at a wavelength of 405 nm to the effective scattering cross sections[2]

[2] The effective scattering cross section is a hypothetical area of a particle that incoming light must impinge in order to be scattered towards the lens. The calibrated effective scattering cross section axis is independent of

and optical diameter[3] of EVs. EVs are modelled as core-shell particles with a core refractive index of 1.38, a shell refractive index of 1.48, and a shell thickness of 6 nm.

Figure 4 shows print screens of the light scatter calibrations.

A

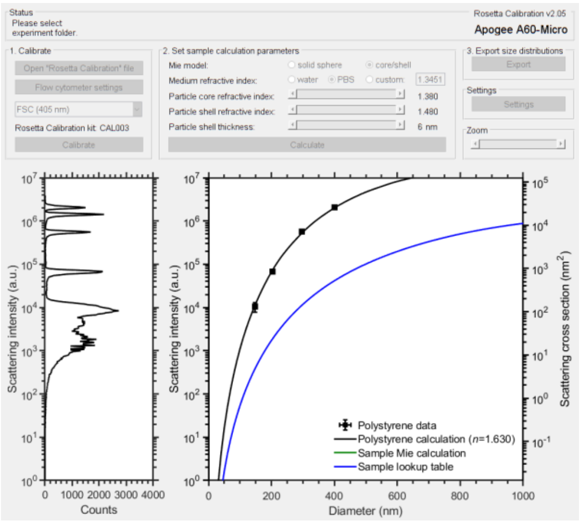

B

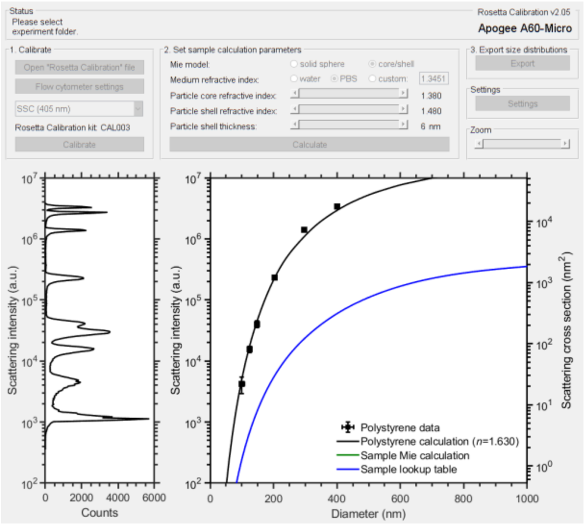

Figure 4. Forward scattering and side scattering calibration of the A60-Micro by Rosetta Calibration. To relate scatter to the diameter of EVs, EVs are modelled as core-shell particles with a core refractive index of 1.38, a shell refractive index of 1.48, and a shell thickness of 6 nm.

7.3.3.2 Flow Scatter Ratio (Flow-SR)

To determine the diameter and refractive index of particles and improve specificity by enabling label-free differentiation between EVs and lipoproteins, the flow scatter ratio (Flow-SR) was applied.

Flow-SR was performed as previously described [5,6]. Lookup tables were calculated for diameters ranging from 10 to 1000 nm, with step sizes of 1 nm, and refractive indices from

---

refractive index assumptions, but depends on the illumination wavelength and collection angles of the flow cytometer [8].

[3] The optical diameter equals the physical diameter of a particle when (1) the particle is spherical and (2) the particle has the same refractive index distribution as assumed in the model.

1.35 to 1.80 with step sizes of 0.001. The diameter and refractive index of each particle was added to the flow cytometry datafiles by custom-build software (MATLAB R2020b).

As Flow-SR requires accurate measurements of both forward scattering and side scattering, we applied Flow-SR only to particles with diameters >200 nm, as determined by Flow-SR, and fulfilling the condition:

$\log_{10} \sigma_{SSC} > -0.8 \cdot \log_{10} \sigma_{FSC} + 2.8$	Equation 2

where σ_{SSC} is the side scattering cross section and σ_{FSC} is the forward scattering cross section.

7.4 Gate description and boundaries

The following gates have been applied to all flow cytometry data files by custom-build software (MATLAB R2020b):

1. During the second that an event was measured, the count rate was within 3.5 standard deviations from the median count rate of the entire measurement, and…
2. the CD235a-PE fluorescence was <10,000 and CD61-BV421 fluorescence was <4000 MESF or the side scattering cross section was <2800 nm^2 to omit cells (only applied to samples stained with CD235a-PE and CD61-BV421), and…
3. the condition in Equation 3 was fulfilled to remove antibody aggregates (applied to CD235a-PE, CD61-BV421 and CD45-APC antibodies, Streptavidin-APC and Lactadherin-FITC, and…
4. the optical diameter is <1,000 nm, and…
    - 4.1. the CD45-APC fluorescence >30 MESF, or…
    - 4.2. the CD235a-PE fluorescence >37 MESF, or…
    - 4.3. the CD61-BV421 fluorescence >45 MESF, or…
    - 4.4. the Streptavidin-APC fluorescence >43 MESF, or…
    - 4.5. the Lactadherin-FITC fluorescence >224 MESF
5. skipping step 4, the diameter, as determined by Flow-SR, is between 200 nm and 650 nm, and…
6. the condition in Equation 2 is fulfilled, and…
    - 6.1. the refractive index, as determined by Flow-SR, is <1.44 to omit false positively labeled lipoproteins, and…
        - 6.1.1. the CD45-APC fluorescence >30 MESF, or…
        - 6.1.2. the CD235a-PE fluorescence >37 MESF, or…
        - 6.1.3. the CD61-BV421 fluorescence >45 MESF, or…
        - 6.1.4. the Streptavidin-APC fluorescence >43 MESF, or…
        - 6.1.5. the Lactadherin-FITC fluorescence >224 MESF
    - 6.2. skipping step 6.1,
        - 6.2.1. the refractive index is <1.44, or…
        - 6.2.2. the refractive index is between 1.45 and 1.70.

$\log_{10} I_{PE} > 0.9x \cdot \log_{10} \sigma_{SSC} - 1.0$	Equation 3

Here, I_{PE} is the fluorescence intensity of the PE detector and σ_{SSC} is the effective side scattering cross section. PDF files with scatter plots of all applied gates are available via the online data repository (section 7.1). The fluorescence gates, which differentiate positively

stained particles from background noise, were automatically determined with a publicly available MATLAB script using a tuning factor of 1.8 [7].

8 References

[1] Théry C, Witwer KW, Aikawa E, Alcaraz MJ, Anderson JD, Andriantsitohaina R, Antoniou A, Arab T, Archer F, Atkin-Smith GK, others. Minimal information for studies of extracellular vesicles 2018 (MISEV2018): a position statement of the International Society for Extracellular Vesicles and update of the MISEV2014 guidelines. *J Extracell Vesicles* 2018; **7**: 1535750.

[2] Lee JA, Spidlen J, Boyce K, Cai J, Crosbie N, Dalphin M, Furlong J, Gasparetto M, Goldberg M, Goralczyk EM, others. MIFlowCyt: the minimum information about a Flow Cytometry Experiment. *Cytom Part A* Wiley Online Library; 2008; **73**: 926–30.

[3] Welsh JA, van der Pol E, Arkesteijn GJA, Bremer M, Brisson A, Coumans F, Dignat-George F, Duggan E, Ghiran I, Giebel B, Görgens A, Hendrix A, Lacroix R, Lannigan J, Libregts SFWM, Lozano-Andrés E, Morales-Kastresana A, Robert S, de Rond L, Tertel T, Tigges J, de Wever O, Yan X, Nieuwland R, Wauben MHM, Nolan JP, Jones JC. MIFlowCyt-EV: a framework for standardized reporting of extracellular vesicle flow cytometry experiments. *J Extracell Vesicles* 2020; **9**: 1713526.

[4] Buntsma NC, Shahsavari M, Gasecka A, Nieuwland R, van Leeuwen TG, van der Pol E. Preventing swarm detection in extracellular vesicle flow cytometry - a clinically applicable procedure. *Res Pract Thromb Haemost* Wiley; 2023; **7**: 100171.

[5] van der Pol E, de Rond L, Coumans FAW, Gool EL, Böing AN, Sturk A, Nieuwland R, van Leeuwen TG. Absolute sizing and label-free identification of extracellular vesicles by flow cytometry. *Nanomed Nanotechnol Biol Med* 2018; **14**: 801–10.

[6] de Rond L, Libregts SFWM, Rikkert LG, Hau CM, van der Pol E, Nieuwland R, van Leeuwen TG, Coumans FAW. Refractive index to evaluate staining specificity of extracellular vesicles by flow cytometry. *J Extracell Vesicles* 2019; **8**: 1643671.

[7] Gankema AAF, Li B, Nieuwland R, van der Pol E. Automated fluorescence gating and size determination reduce variation in measured concentration of extracellular vesicles by flow cytometry. *Cytom Part A* Wiley Online Library; 2022; **101**: 1049–56.

[8] Welsh JA, Arkesteijn GJA, Bremer M, Cimorelli M, Dignat-George F, Giebel B, Görgens A, Hendrix A, Kuiper M, Lacroix R, others. A compendium of single extracellular vesicle flow cytometry. *J Extracell Vesicles* Wiley Online Library; 2023; **12**: e12299.
